# Understanding women’s, caregivers’, and providers’ experiences with home-based records: A systematic review of qualitative studies

**DOI:** 10.1371/journal.pone.0204966

**Published:** 2018-10-04

**Authors:** Olivia Magwood, Victoire Kpadé, Ruh Afza, Chinedu Oraka, Jennifer McWhirter, Sandy Oliver, Kevin Pottie

**Affiliations:** 1 C.T. Lamont Primary Health Care Research Centre, Bruyère Research Institute, Ottawa, Canada; 2 University of Ottawa, Ottawa, Canada; 3 Department of Population Medicine, University of Guelph, Guelph, Canada; 4 Department of Social Science, University College London, London, United Kingdom; 5 University of Johannesburg, Johannesburg, South Africa; 6 Departments of Family Medicine & Epidemiology and Community Medicine, University of Ottawa, Ottawa, Canada; Aga Khan University, KENYA

## Abstract

Mothers, caregivers, and healthcare providers in 163 countries have used paper and electronic home-based records (HBRs) to facilitate primary care visit. These standardized records have the potential to empower women, improve the quality of care for mothers and children and reduce health inequities. This review examines experiences of women, caregivers and providers with home-based records for maternal and child health and seeks to explore the feasibility, acceptability, affordability and equity of these interventions. We systematically searched MEDLINE, MEDLINE In-Process, MEDLINE Ahead of Print, Embase, CINAHL, ERIC, and PsycINFO for articles that were published between January 1992 and December 2017. We used the CASP checklist to assess study quality, a framework analysis to support synthesis, and GRADE-CERQual to assess the confidence in the key findings. Of 7,904 citations, 19 studies met our inclusion criteria. In these studies, mothers, caregivers and children shared HBR experiences in relation to maternal and child health which facilitated the monitoring of immunisations and child growth and development. Participants’ reports of HBRs acting as a point of commonality between patient and provider offer an explanation for their perceptions of improved communication and patient-centered care, and enhanced engagement and empowerment during pregnancy and childcare. Healthcare providers and nurses reported that the home-based record increased their feeling of connection with their patients. Although there were concerns around electronic records and confidentiality, there were no specific concerns reported for paper records. Mothers and other caregivers see home based records as having a pivotal role in facilitating primary care visits and enhancing healthcare for their families. The records’ potential could be limited by users concerns over confidentiality of electronic home-based records, or shortcomings in their design. Health systems should seize the opportunity HBRs provide in empowering women, especially in the contexts of lower literacy levels and weak health care delivery systems.

## Introduction

The home-based record (HBR) offers an approach that women and countries can use to improve both the processes, such as communication and empowerment, and outcomes of health care, including pregnancy complications, child development and vaccination [1]. The HBR is a document that may include components of preventive or curative antenatal, postnatal, newborn, and child health. This type of record has been used in various paper or electronic formats since the introduction of the Japanese Maternal and Child Health Handbook in 1948 [2]. Today, over 163 countries have used HBRs [3]. New card designs and delivery approaches that span the spectrum of care, from pregnancy through to childhood, offer opportunities for countries that wish to enhance the continuity of care and reduce child and maternal mortality.

United Nation (UN) Sustainable Development Goals 3 and 5 aim to reduce the mortality rates of children under age 5 and improve maternal health by the year 2030 [4]. A pivotal component of Goal 5 is the realization of gender equality and the empowerment of women. Disempowerment is associated with poorer health and social outcomes for women and children [5]. Inequities in gender, age, socioeconomic status and ethnicity contribute to disempowerment [6, 7]. Empowerment is both a process and an outcome that allows individuals to take control over their lives, set their own agendas, gain skills, increase self-confidence, solve problems, and develop self-reliance [8].

To date, there is no global synthesis of evidence that incorporates the perceptions of caregivers and mothers in relation to these HBRs. Hence, the objective of this study is to examine and synthesize existing published research about mothers, caregivers, children and health care providers in terms of their use and acceptability of HBRs, and the value of using these records. This systematic review is one of a series of systematic reviews commissioned by the WHO to underpin forthcoming global guidance on home-based records for maternal, newborn and child health. Other reviews in the WHO series examine the effectiveness of HBRs on health outcomes [1].

HBRs are designed for use in primary and secondary-care encounters [9]. HBRs aim to bridge patients and providers; however, this is dependent on local feasibility, acceptability, applicability, and their value, such as vaccine-series completion and child-growth monitoring. Women who engage with these interventions are more likely to participate in primary care and to ensure the continuity of care [10]. The WHO and the United Nations Children's Fund’s (UNICEF) Expanded Program on Immunisation (EPI) have supported cultural and language adaptations to HBRs, but evaluations are needed to assess the benefits and harms of HBRs [3]. To improve the implementation of HBRs, it is important to assess the perspectives of mothers, caregivers, and providers, and also to determine how these may vary across rural and urban areas, and private and public clinics in low-, middle- and high-income countries.

Electronic HBRs have begun to be used in middle- and high-income settings [11]. The use of this type of record prevents data loss and promotes information sharing between providers to improve integration in care [12]. Part of our review aims to compare paper-based HBRs to the newly emerging electronic records and looks at how women and caregivers perceive these electronic HBRs in terms of their value, security and ease of use. For health equity concerns, we aim to consider low-literacy populations and populations that do not speak their home country’s official language, as well as mobile populations, such as nomads, internally displaced persons, and refugees.

To achieve our study objective, this systematic review addresses the following key research question: Are HBRs for maternal, newborn and child health feasible, acceptable, affordable and equitable from the perspectives of women, family members, and health provider stakeholders? This review also aims to understand the values that women and caregivers hold in relation to the use of these HBRs.

## Methods

We searched for qualitative studies exploring the experiences of mothers, caregivers and healthcare providers with home-based records for maternal, newborn and child health. We utilized the best fit framework analysis method for the synthesis of this systematic review [13]. We selected a framework *a priori* and searched for constructs of acceptability, feasibility, affordability and equity as defined by the Grading of Recommendations Assessment, Development and Evaluation (GRADE) [14]. We identified qualitative key findings and assessed the confidence of the key findings using GRADE-CERQual [15, 16].

### Search strategy and selection criteria

This systematic review adheres to the Preferred Reporting Items for Systematic Reviews and Meta-Analyses (PRISMA) guidelines [17]. A team of experts developed a protocol that considered the use, implementation and values that are relevant to mothers, caregivers, and healthcare provider stakeholders in low-, middle- and high-income countries, in relation to the use of paper and electronic HBRs, which was published on the Cochrane Equity Methods website [18]. Using relevant search terms, searches of MEDLINE, MEDLINE In-Process, MEDLINE Ahead of Print, Embase, CINAHL, ERIC, and PsycINFO accessed articles that were published between January 1992 and August 2017. The search strategy is listed in Supplemental 1 (S1). We also searched the grey literature to identify relevant studies and published reports on prevention programmes of the World Health Organization (WHO), the Centre for Disease Control and Prevention (CDC), the European Centre for Disease Prevention and Control, the United States Agency for International Development, John Snow Inc. (JSI), and the Japan International Cooperation Agency (JICA).

We included qualitative and mixed-methods studies that reported on the values of and perceptions around HBRs and their access, use, feasibility, affordability, equity and acceptability. We focused on studies on mothers, caregivers, children and healthcare stakeholders and considered low-, middle- and high-income settings. Papers were eligible for inclusion if they addressed the research question, utilized qualitative methods, and included qualitative evidence (See Supplemental 2 (S2) for the full inclusion and exclusion criteria). These reports could be in any language or geographic setting.

### Study selection and data extraction

An independent team screened titles and abstracts in duplicate, followed by full-text assessments for eligibility. Conflicts were resolved through discussion or the involvement of another reviewer. Citation information was downloaded into EndNote reference software. We assessed the methodological quality of papers using the U.K Critical Appraisal Skills Programme (CASP) checklist for qualitative studies [19]. While we used CASP to assess the quality of all included studies, we did not exclude any papers on the basis of quality assessment, rather, the methodological rigor of each contributing study contributed to the confidence assessments of each review finding.

We designed our data-extraction form according to a framework selected *a priori*: the social-ecological model (See Table 1) for behaviour change [20, 21]; which has been used in previous research that explores maternal and child health [22, 23]. This approach facilitated the exploration of maternal, caregiver and health care provider experiences with HBRs. The social-ecological model is a theory-based framework that considers the complex interconnections of the multiple levels of a social system and the interactions between individuals and their environment [22]. Understanding how HBRs influence social ecology, defined as the study of the relation between the developing human being and the settings and contexts in which the person is actively involved [24], allows for the investigation of acceptability and usability of HBRs at multiple levels of a social system. Our data-extraction form reflects the model’s system levels, which include the individual, interpersonal and family, community and social, and organizational and policy levels. Within each of these levels, we examined the determinants of HBR use, acceptability, feasibility, affordability and equity. We pilot tested the data-extraction form to ensure the framework aligned with the data. Our team of reviewers extracted data, in duplicate, from the included studies. Discrepancies were resolved through discussion.

**Table 1:** Description of social ecological model framework levels.

Framework level	Description	Examples
**Individual**	Individual Characteristics of an individual that influence behaviour change, including knowledge, attitudes, behaviour, self- efficacy, developmental history, gender, age, religious identity, racial/ethnic/caste identity, sexual orientation, socio-economic status, financial resources, values, goals, expectations, literacy, stigma, and others.	Age and education, knowledge and need of maternal health care, mistrust, low decision- making autonomy, financial burden, risk perception
**Interpersonal or family**	Formal (and informal) social networks and social support systems that can influence individual behaviours, including family, friends, peers, co-workers, religious networks, customs or traditions.	Family tradition, husbands knowledge and perceptions, influence of mothers-in-law or other relatives
**Community and social**	Relationships among organizations, institutions, and informational networks within defined boundaries, including the built environment (e.g. parks), village associations, community leaders, business and transportation.	Influence of Community health workers, poverty, religious belief, traditional practices (ex: delivery and breastfeeding practices), influence of neighbours, gender norms, health beliefs Organizational and health system
**Organizational and health system**	Organizations or social institutions with rules and regulations for operations that affect how, or how well, MNCH services are provided to an individual or group.	Availability of services, behaviour/quality of healthcare providers, accessibility (distance, cost).

### Data synthesis

We contextualized the preliminary findings on HBRs and maternal, newborn and child populations, using the social-ecological framework [21]. We used the framework method as a systematic and flexible approach to analysing qualitative data [25] and grouped ideas of acceptability, feasibility, affordability and equity across key populations. Framework analysis is a five stage process of familiarisation with the data, identifying a thematic framework, indexing (applying the framework), charting and mapping, and interpretation [26]. Any relevant data that did not correspond to the components of our framework were incorporated as emerging themes. This coding was done in a matrix spreadsheet to facilitate analysis. Mapping involved examining concordant findings, disconfirmatory data, and associations between themes. Interpretations were guided by our review objectives as well as emerging themes.

We applied a qualitative methods lens that considered the saturation level (no new themes revealed in examining new papers) and the triangulation of the data between the mothers, caregivers, stakeholders and organizations within the health systems in the study. In judging the relevance to our research question, we considered the design of the HBR (for example, an integrated maternal and child record), the setting and the outcome. We used the data contained in the framework analysis to identify the key findings on the themes of feasibility, acceptability, affordability and equity. A key finding is defined as a synthesis of qualitative evidence that describes a recurring phenomenon found in primary studies [15, 16].

We used the Confidence in the Evidence from Reviews of Qualitative research (CERQual) tool [15] to assess the confidence of the key findings of this review. This tool is a new method used for assessing the strength of qualitative review evidence; it works similar to the way the Grading of Recommendations Assessment, Development and Evaluation (GRADE) approach assesses the strength of quantitative evidence [14]. CERQual bases the evaluation on four criteria: the methodological limitations of the included studies that support a review finding; the relevance of the included studies to the review question; the coherence of the review findings; and the adequacy of the data that contributes to a review finding The GRADE-CERQual assessment results in a final classification of confidence in the theme in four categories: ‘high’, ‘moderate’, ‘low’ or ‘very low’ (See Tables 2 and 3).

**Table 2:** CERQual assessment component.

Component	Definition
Methodological limitations	The extent to which problems were identified in the way in which the primary studies which contributed to the evidence for a review finding were conducted
Relevance	The extent to which the primary studies supporting a review finding are applicable to the context specified in the review question
Coherence	The extent to which the pattern that constitutes a review finding is based on data that is similar across multiple individual studies and/or incorporates (compelling) explanations for any variations across individual studies
Adequacy of data	An overall determination of the degree of richness and/or scope of the evidence and quantity of data supporting a review finding

**Table 3:** Definitions of levels of confidence in the CERQual approach.

Level	Definition
High confidence	It is highly likely that the review finding is a reasonable representation of the phenomenon of interest
Moderate confidence	It is likely that the review finding is a reasonable representation of the phenomenon of interest
Low confidence	It is possible that the review finding is a reasonable representation of the phenomenon of interest The review finding may be a reasonable representation of the phenomenon of interest
Very low	It is not clear whether the review finding is a reasonable representation of the phenomenon of interest It is not clear/We are uncertain whether the review finding is a reasonable representation of the phenomenon of interest

## Results

**Fig 1:**
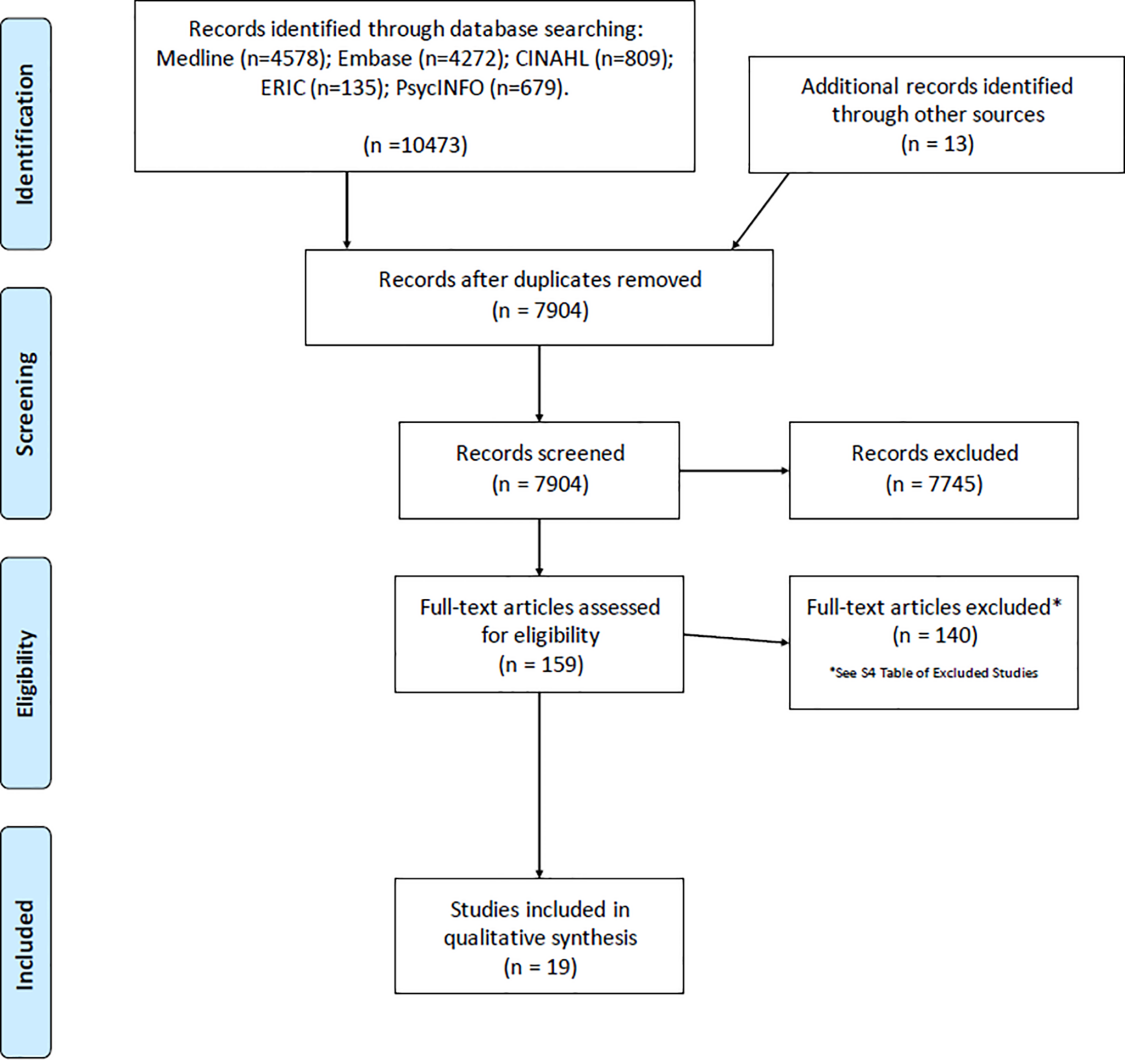
PRISMA flow diagram.

Our search strategy identified 10,486 citations. After removing the duplicates, we screened 7,904 articles by title and abstract. We went on to screen 159 articles, using a full-text assessment for eligibility. Fig 1 shows the 19 studies that met our inclusion criteria.

Table 1 shows the characteristics of the included studies. These studies are heterogeneous in terms of sample size, home-based record design, setting and findings. Of the 19 included studies, four were set in low- or middle-income countries (Brazil, Palestine, South Africa, and Cambodia). The remaining 15 studies took place in the UK (5), the US (5), Australia (3), Canada (1), and New Zealand (1). Interventions included child health books (9), online child health portals (4), the Maternal and Child Health Handbook (2), women-held antenatal records (2), online antenatal records (1), and electronic child immunisation records (1). The majority of the included studies used qualitative techniques, and most data were collected by individual interviews and/or surveys. They represented the views of more than 2700 pregnant women, mothers, caregivers, and healthcare providers. The CASP summary of methodological assessment is also included in Table 4.

**Table 4:** Characteristics of included studies.

Reference	Country	Study Design	Population	Intervention	Focus of the Study	CASP Quality Assessment
Byczkowski, 2014	USA	Mixed methods: Cross-sectional telephone survey with semi-structured interviews	N = 530 parents and caregivers; 215 intervention users, 315 non-users for telephone survey, and 126 of the 215 portal users for the survey	A secure web-based portal through which parents can access laboratory results, medication information, and their child’s visit history	Measures and understands parent concerns and perceptions of the usability and value of using a web-based portal to access their child's health record	8/10
Clendon, 2010	New Zealand	Oral History	N = 35 participants using the intervention	Child health and development record books	Examines the role and impact of the child health and development record book in New Zealand society and its inceptions	10/10
Grippo, 2008	Brazil	Descriptive study	N = 89 family caregivers responsible for 0–59 month-old children	Booklet that presents topics related to children's development, including pregnancy and raising children healthy	Evaluates the effectiveness, identifies people's acceptance, characterizes family comprehension, and analyses relatives’ perceptions of child development and pregnancy booklet	7/10
Hagiwara, 2013	Palestine	Mixed methods: Cross sectional study with focus-group discussions	N = 67 participants: 42 women and 25 health professionals from the intervention areas	MCH handbook that monitors health of women, surveys use of health services, promotes health education, provides info when mother or child is referred	Evaluates the impact, satisfaction, and constraints of using the maternal child health handbook	8/10
Hamilton, 2012	Australia	Mixed methods: Online survey with open-ended questions and semi-structured interview	N = 120 mothers did an online questionnaire; 6 mothers participated in interviews	Child Personal Health Record	Evaluates the effects of parent use of child personal health records on the parents’ experience, knowledge, engagement with child care	9/10
Harrison, 1998	South Africa	Descriptive prospective study	N = 185 interviews of 35 health personnel and 150 mothers/caregivers	Revised version of the Road-to-Health card. It now contains a weight-for-age-chart, immunisation schedules and other health related data	Describes the opinions of health personnel and parents on the accuracy and completeness of data recorded on the Road-to-Health card, and the information they would like recorded	8/10
Hill, 2003	Scotland	Mixed methods. Self-completion questionnaires were used for data collection	N = 871 participants: 12 health professionals, 749 children, 100 parents and 10 teachers	Child Health record	Determines the views of children, parents, teachers and health professionals on the Child Health profiles, and suggestions improvements	8/10
Hully, 1993	England	Semi-structured questionnaire	N = 18 parents of children from the paediatric oncology unit	Parent-held records for children	Explores the efficiency of the patient held record	9/10
Hunter, 2008	Scotland	Semi-structured face-to-face interviews	N = 12 Residential Care Workers	The BAAF common documentation form	Explores why the the shared documentation was not used routinely and the perceptions of residential care workers in their role of health improvement	10/10
Kelly, 2016	USA	Cross-sectional study	N = 90 parents	Online portal for parents of children	Assesses parent use and perceptions of an inpatient portal application that provides information about a child’s hospital stay	9/10
King, 2017	Canada	Prospective, mixed-methods study	N = 23 participants: 18 caregivers, 5 service providers	Connect2care online health portal.	Examines the use, utility, and impact of the connect2care portal	9/10
Kitayama, 2014	USA	Focus groups	N = 29 parents.	Online immunization record for pediatric patients	Examines desired characteristics of an online immunization record for parents	9/10
Lee, 2016	USA	A qualitative evaluation	N = 40 families: 20 in each phase	The pediatric patient passport program	Evaluates the impact of a patient–provider communication program, The Patient Passport Program, to improve the health care experience and satisfaction of culturally diverse families of hospitalized children	8/10
O’Connor, 2016	England	Exploratory case study	N = 33 participants: 12 parents and 10 health visitors	Personal child health record	Illuminate the factors that hindered Health Visitors in engaging parents to use the eRedBook in order to improve how the personal child health record is implemented	7/10
Phipps, 2001	Australia	Interviews	N = 21 in their 2nd or 3rd trimester who attended an antenatal clinic at least twice	Women held antenatal card,	Explores whether women perceived carrying their own medical records would beneficial	10/10
Quinlivan, 2014	Australia	Mixed methods	N = 474 obstetric patients	Women’s Personally Controlled Electronic Health Record	To survey antenatal patients to determine their preferred medical record system	8/10
Sharp, 2014	USA	Descriptive study	N = 4 childhood cancer survivors, 11 caregivers of younger cancer survivors, and 5 survivor–caregivers	Pediatric Electronic Personal Health Record	Explores the knowledge, interest, and attitudes of a sample of survivors and some of their caregivers towards electronic personal health records	10/10
Whitford, 2014	Scotland	An exploratory, qualitative, longitudinal study	N = 95 participants: 42 women participated in antenatal interviews completed postnatal interviews and 24 health professionals	Scottish Woman-Held Maternity Record	To investigate women's and staff's experiences with a standard birth plan, integral to a maternity record and to investigate how opportunities for women to co-construct maternity records could contribute to the provision of women-centered care	8/10
Yanagisawa, 2015	Cambodia	Mixed-methods: Pre and post intervention surveys	N = 38 participants: 20 multiparous women, 8 midwives & nurse, 10 VHV & TBAs	MCH Handbook	Assesses the cultural appropriateness of the MCH handbook and explored the potential obstacles and effects associated with its implementation	9/10

Findings were grouped according to the constructs of feasibility, acceptability, affordability and equity. The study findings were categorised into individual, interpersonal and family, community and social, organizational and health system levels of the SEM framework. From synthesising descriptions from included studies, we identified three broad types of HBRs used by mothers or caregivers: maternal health records, child health records, and immunisation records. The differences among these interventions played a role in the perceptions of mothers, caregivers and healthcare providers of the value of HBRs for maternal, newborn and child health. We categorised the emerging findings according to the intervention used (See Table 5).

**Table 5:** Framework analysis.

	Main Theme (finding)	Studies Cited	Intervention-Specific Supporting Evidence	Illustrative Quotes
**Individual** *Characteristics of an individual that influence behaviour change*, *including knowledge*, *attitudes*, *behaviour*, *self-efficacy*, *developmental history*, *gender*, *age*, *religious identity*, *racial/ethnic/caste identity*, *sexual orientation*, *socioeconomic status*, *financial resources*, *values*, *goals*, *expectations*, *literacy*, *stigma*, *and others*.	Home-based records improve the knowledge of mothers and help them share in pregnancy decision making, and improve caregiver’s knowledge about their child’s health status.	7 (Phipps 2001, Yanagisawa 2015, Byczkowski 2014, Kelly 2017, Lee 2016, Kitayama 2014, Whitford 2014)	Maternal Health Record • Having extra knowledge made them feel more involved and helped them share in decision making (Phipps 2001) • The MCH handbook was associated with increased healthcare knowledge in mothers (Yanagisawa 2015) Child Health Record • Majority of parents agreed that they better understood their child's condition (Byczkowski 2014) • Parents love the fact that it kept them informed about their child's health (Kelly 2017) • Parents who had access to an online portal suggested that more educational resources and information be available (Byczkowski 2014) Immunisation Record • The ability to identify whether or not their child’s immunizations were up-to-date was also highlighted. (Kitayama 2014)	Maternal Health Record • *“It was really quite good*, *because there was things [*.* *.* *.*] there’s stuff I suppose that you wouldn’t even think of*, *unless you went through that*.*”* (Whitford 2014) Child Health Record • *“Love the fact that it kept me informed about my child’s health”* (Kelly, 2017) • *Language/improved communication—“What made the care better was I entered the passport Program and then I could understand everything inside of it”*. (Lee 2016) Immunisation record • *“If they are missing any vaccinations*, *how many they’ve had… Always is it up to date because usually they need booster shots or whatever or they’re missing a vaccination”* (Kitayama, 2014)
	The use of home-based records for maternal and child health decrease fear among users and improve confidence and feelings of empowerment during patient-provider interactions.	8 (Clendon 2010, Grippo 2008, Quinlivan 2014, Whitford 2014, Hamilton 2012, Hully 1993, Lee 2016, Sharp 2014)	Maternal Health Record • The book is used as a practical tool of motherhood but also reflects to the mother her actions in raising a child thus contributing to her identity in the role (Clendon 2010) • It serves as a proposal to discuss and favour empowerment. (Grippo 2008) • Women improve their sense of control and satisfaction when they own their maternal records (Hagiwara 2013) • Feelings of control using personal records: "I can control who sees it" (Quinlivan 2014) • Staff noted that explicit reference to birth plans could reassure women (Whitford 2014) Child Health Record • Parents interacted more closely with the CPHR for their 1st born child. After that they got busy and/or became more comfortable and confident with parenting—CPHR has the potential to provide a context of parent empowerment (Hamilton 2012) • Helped parents concentrate on the needs of the child, rather than having to remember when they were last given treatment (Hully 1993) • Mothers felt less fearful, more comfortable and empowered to ask questions (Lee 2016) • The most common perceived benefit related to confidence that the health information would not be lost or that it would be easier to find because of a centralized location (Sharp 2014)	Maternal Health Record: • *“Because I’m able to write all that down in there*, *I feel much more at ease going in”* (Whitford 2014) • *“I guess if it’s on a computer file it can’t get lost*. *My hospital record got lost once*. *But they did find it eventually*.*”* (Quinlivan 2014) Child Health Record: • Decrease fear: *“I think the Passport opened up a lot of doors—because I had someone beside me*, *too*, *advocating with me*.*”* (Lee 2016) • Improved confidence: “*You gain confidence as a parent and you stop referencing stuff as much … my reduced usage of the CPHR corresponds with my reduced usage of all sources of information”* (Hamilton, 2012) • *“[The book] is to empower the parent and to reinforce any strengths or positive parenting”* (Clendon 2010) • *“I can control who sees it*.*”* (Quinlivan 2014)
	Across all types of home-based records, mothers and caregivers had concerns with the privacy of electronic records.	4 (Kitayama 2014, O’Connor 2016, Quinlivan 2014, Sharp 2014)	Maternal Health Record • Fear of government intervention and lack of privacy once records are online (Quinlivan 2014) Child Health Record • HVs and parents had concerns over the security of children’s health data if it is was going to be held by a private multinational company and not governed by the NHS (O’Connor 2016) • Among those who offered concerns, data security and privacy were the most common (Sharp 2014) Immunisation Record • Serious concerns with protecting privacy, especially regarding their children’s medical information, surfaced in all 4 focus groups (Kitayama 2014)	Maternal Health Record • *"They say that only you can see it*, *but in a few years that will change*. *All those politicians will want to ransack our records for things and you won't get a say in how they use them*. *Once somethings online you've lost control*.*"* (Quinlivan 2014) • *“If I have it then no one else can see it unless I show it to them*.*”* (Quinlivan 2014) • *“You hear about people breaking into computers and stealing information*. *You know*, *like Wikileaks*, *only they just want to cause trouble*. *I’m not sure I want all my medical information out there to be discovered*. *Who reads it*? *I also don’t want my husband or kids seeing things either and if its [sic] there they might want to see*. *I’m not convinced it would be safe*.*”* (Quinlivan 2014) Immunisation Record • *“I have a suggestion*. *A lot of immigrant parents are scared*. *It should be noted on here that it will be confidential*. *That they should not worry about*, *you know*, *their status”* (Kitayama, 2014) . Child Health Record: • *“I know what a personal health record is and I would not want my child to have one—period*. *I am concerned with all types of hackers and the information they can get”* (Sharp 2014).
**Interpersonal/Family** *Formal (and informal) social networks and social support systems that can influence individual behaviours*, *including family*, *friends*, *peers*, *co-workers*, *religious networks*, *customs or traditions*.	The use of home-based records for maternal health increased husbands involvement with pregnancies and helped deal with misconceptions about pregnancy that other family members believed.	3 (Hagiwara 2013, Phipps 2001, Yanagisawa 2015)	Maternal Health Record • Sharing the records with their partner or husband and being able to go through the records was a consistent positive theme. When the husband worked long hours and was unable to be involved in antenatal care, reading the records enabled him to feel closer and more engaged in the pregnancy.—Records contained information that fostered communication within the relationship. Women enjoyed sharing the information with people like grandparents and friends. (Phipps 2001) • Some husbands showed interest and commented that the book was useful and contained meaningful illustrations. Some husbands explained the contents of the handbook to their wives and advised them to obtain ANC, avoid salty food, or refrain from working too hard. (Yanagisawa 2015) • Husband's participation in pregnancy care, delivery planning, and child care improved after receiving the MCH handbook because he had a chance to read it at home—Education messages included in the MCH handbook were effective in dealing with rumors and misconceptions about pregnancy care and childcare that they, their family members and especially their mothers-in-law believed. (Hagiwara 2013)	Maternal and Child Health Record: • Authors stated the MCH handbook helped mothers/caregivers deal with rumours and misconceptions about pregnancy and childcare, their families and especially their mother-in-laws (Hagiwara 2017).
	The use of home-based records for child health improved family engagement with child care.	3 (Clendon 2010, Grippo 2008, King, 2017)	Child Health Record • The Plunket book is an important artifact that is kept and reflected upon for years. The older mother takes on a new mothering role offering care and support as her child transitions to motherhood (Clendon 2010) • Family attitudes were recognized as relevant to basic care for children health development (Grippo 2008) • The portal provided a positive, inviting message to families about being engaged. The service providers saw the utility of the portal in setting up appointments and providing secure messaging to the families. (King 2017)	Child Health Record • The portal provided a positive, inviting message to families about being engaged (King 2017).
**Social/Community** *Relationships among organisations*, *institutions*, *and informational networks within defined boundaries*, *including the built environment (e*.*g*. *parks)*, *village associations*, *community leaders*, *business and transportation*.	The use of home-based records for maternal and child health facilitated communication between mothers/caregivers and healthcare professionals and improved person-centered care.	12 (Byczkowski 2014, Clendon 2010, Grippo 2008, Hagiwara, 2013, Hamilton 2012, Hunter 2008, Hully 1993, Lee 2016, King 2017, Phipps 2001, Quinlivan 2014, Sharp 2014, Whitford 2014)	Maternal Health Record • Women revealed that it was easier to ask questions of health providers when they referred to the MCH handbook. (Hagiwara, 2013) • Carrying their own records encouraged health care workers to better explain what was being recorded and why certain things were done, as they were aware women would go home and read the records again. (Phipps 2001) • Patients had fewer concerns with poor communication with health staff with personal records vs hospital held record (Quinlivan 2014) • Both midwives and doctors mentioned that the birth plan could support useful discussions with women both during pregnancy and labor. Antenatally, it could prompt and guide conversations about labor and birth options (Whitford 2014). Child Health Record • Majority of parents agreed that it improved communication with healthcare providers and their ability to manage the condition (Byczkowski 2014) • For mothers, the book was useful as a tool to facilitate establishing a relationship with their nurse (Clendon 2010) • Most caregivers reported that the booklet served as strengthening elements in the relationship with the health unit's professionals. (Grippo 2008) • CPHRs had the potential to empower parents to communicate their views regarding their children's health (Hamilton 2012) • It was useful as a memory aid for asking questions at clinic visits, and kept a lot of information together (Hunter 2008) • All clinicians reported improved communication with patients and their families, as well as an increase in the types of questions asked by family members. (Lee 2016) • It was useful as a memory aid for communicating at clinic visits (Hully 1993) Participants expressed an appreciation for having more detailed information and knowing the technical language, as they felt they could then communicate on a more level playing field with providers. The portal showed potential in enhancing engagement in care and communication with care providers (King 2017)	Maternal Health Record: • *“It’s good as I showed my card to the midwife and she gave me my injection for the blood ([sic] anti-D)*. *Otherwise I would have had to wait to get everything checked again*.*”* Child Health Record: • *“[if] we can use accurate language*, *language they understand*, *we get a lot better dynamic where they’re going to listen to us”* (King 2017) • *“messages of we want you to be engaged*, *we’re trying to reach out to you*, *we’re trying to have multiple ways of engaging with you”* (King 2017) • Overall understanding—*“”I feel very proud that they really care about my thinking”* (Lee 2016) • *“I could read what effect the meds have because I could read this info in Spanish*, *because they were giving me the info in Spanish”*. (Lee 2016) • “*It would be good because everything is together*. *I could print things out for the doctor and the communication would be faster*. *Also*, *it would provide information for my daughter when she gets older to share with teachers and doctors”* (Sharp 2014) • *“I found the book worked really well*, *that it was like a communication between the both of you”* (Clendon 2010)
	Home-based records acted as a point of commonality between caregivers/mothers and nurses, and allowed nurses to provide more comprehensive/tailored health education.	6 (Hagiwara 2013, Lee 2016, Yanagisawa 2015, Clendon 2010, Hamilton 2012)	Maternal Health Record • Health providers expressed that they could provide more comprehensive health education and counseling with greater confidence and accuracy, when they used the MCH handbook—they feel committed and relaxed in providing all the necessary information related to MNCH care with the guidance of MCH handbook (Hagiwara, 2013) • Like the mothers, all of the health centre staff, VHVs and TBAs preferred the MCH handbook to the current record system in terms of its appearance, information, convenience and long-term value. All of them used the handbook for health education. (Yanagisawa 2015) Child Health Record • For nurses the Plunket book is useful as a clinical tool to guide interventions and track progress with the mother over time and a tool that could be used to identify and build on the strengths in a family, encouraging positive interactions between family members and healthcare professionals (Clendon 2010) • Nurses more than physicians felt the Passport was more useful, that it increased their feeling of connection with the family, and that it helped them provide culturally sensitive care (Lee, 2016) • CPHR was a vehicle for sharing information between healthcare professionals (Hamilton 2012)	Maternal Health Record • Health providers expressed that they could provide more comprehensive health education and counseling with great (Hagiwara, 2013) Child Health Record • “[the book] was like a stepping stone between the both of you” (Clendon 2010)
**Organizational/Health System** *Organizations or social institutions with rules and regulations for operations that affect how*, *or how well*, *MNCH services are provided to an individual or group*.	The use of home-based records for maternal and child health facilitated continuity of care.	3 (Hamilton 2012, Hully 1993, King 2017, Quinlivan 2014)	Child Health Record • CPHR facilitated continuity of care and served as a vehicle for sharing information between health care professionals (Hamilton 2012). • Caregivers commented on the utility of cross-organization EHRs, connection to the adult health care system, provision of information about available programs, and personalization on a broad scale (King 2017). • Parents of children in an oncology unit believed that a personal health record helped to ensure continuity of care (Hully 1993).	Maternal Health Record • *“I think it would help my GP know what the hospital were doing and stop tests being repeated*.*”* (Quinlivan 2014)
	Healthcare providers value the educational and logistical aspect of home-based records.	5 (Hagiwara 2013, Phipps 2001, Lee 2016, King 2017, Harrison 1998)	Maternal Health Record • Health providers expressed the opinion that they could provide more comprehensive health education and counseling, with greater confidence and accuracy, when they used the MCH handbook (Hagiwara 2013) • The fact that women carrying their own records encouraged health care workers to better explain what was being recorded and why certain things were done, as they were aware women would go home and read the records again (Phipps 2001). • One clinician noted that the clearly delineated time for rounding allowed family members to think through their questions and to be well prepared for their meetings (Lee 2016). • Service providers saw the utility of the portal in setting up appointments and providing secure messaging (King 2017). • All nurses interviewed felt that the RTH card should play an educational role (Harrison 1998).	Child Health Record • Authors stated that nurses valued the child health book because it connected them to families and helped them provide culturally appropriate care (Lee, 2016).
	A study in a low income setting reports that women value the ease, speed and convenience of online home-based records. Healthcare practitioners from two low income countries report that they value the design of home-based records.	3 (Kitayama 2014, Yanagisawa 2015, Harrison 1998)	Online immunization record • Parents expressed that the main advantage of a personal online application was the relative ease, speed, and convenience with which they could access their child’s updated immunization record for school forms and other paperwork (Kitayama 2014). Maternal Health Record • Like the mothers, all of the health centre staff, VHVs and TBAs preferred the MCH handbook to the current record system in terms of its appearance, information, convenience and long-term value (Yanagisawa 2015). Child Health Record • Clinical staff supported the concept of the card but not its composition… The original was designed and implemented without their advice and it does not meet their needs. All those interviewed favoured a more personal document containing nutritional data, illustrated milestones, a simple weights chart, a more comprehensive immunisation schedule, information regarding the treatment of common problems and space for clinic staff and mothers to take notes (Harrison 1998).	Online immunization record • *"You can do a lot of things automatically*. *It saves a lot of time"* (Kitayama, 2014).

Positive experiences with HBRs emerged as a composite outcome of our results. We identified ten key findings and assessed the confidence in these findings, using GRADE-CERQual (See Table 6). Confidence in findings ranged from very low to low. Confidence levels were downgraded due to the methodological limitations, the relevance to the setting, and the coherence and adequacy of the data.

**Table 6:** CERQual summary of findings.

Review Finding	CERQual Assessment of Confidence in the Evidence	Explanation of CERQual Assessment	Studies Contributing to the Review Finding
**Home-based records improve the knowledge of mothers and help them share in pregnancy decision making, and improve caregiver’s knowledge about their child’s health status.** Illustrative Quote: “Love the fact that [the child health record] kept me informed about my child’s health” (Kelly, 2017).	Low confidence	Knowledge consistently reported benefit for records even across a range of record styles. The major concern came with variance in record design (relevance), and the adequacy of the data, in that many studies did not show rich data, saturation or member checking.	Phipps 2001, Yanagisawa 2015, Byczkowski 2014, Kelly 2017, Lee 2016, Kitayama 2014, Whitford 2014
**The use of home-based records for maternal and child health facilitated communication between mothers/caregivers and healthcare professionals and improved person-centered care**. Illustrative Quote: “I found the book worked really well, that it was like a communication between the both of you” (Clendon 2010).	Low confidence	The major concerns were with the relevance of the finding and its adequacy because of the limited number of participants in studies.	Byczkowski 2014, Clendon 2010, Grippo 2008, Hagiwara, 2013, Hamilton 2012, Hunter 2008, Hully 1993, Lee 2016, King 2017, Phipps 2001, Quinlivan 2014, Sharp 2014, Whitford 2014
**The use of home-based records for maternal and child health decrease fear among users and improve confidence and feelings of empowerment during patient-provider interactions.** Illustrative Quote: “I think the Passport [health record] opened up a lot of doors” (Lee 2016). “I can control who sees it.” (Quinlivan 2014).	Low confidence	Across a variety of record types, increase in confidence and decrease in fear were consistently reported. The major concerns revolved around the setting limitation and the overall richness of data.	Clendon 2010, Grippo 2008, Quinlivan 2014, Whitford 2014, Hamilton 2012, Hully 1993, Lee 2016, Sharp 2014
**Mothers and caregivers had concerns with the privacy of online or electronic health records.** Illustrative Quote: “I’m not sure I want all my medical information out there to be discovered. [. . .] I’m not convinced it would be safe.” (Quinlivan 2014).	Low confidence	Fear of privacy reported inconsistently in 1 study. Relevancy of settings is a concern as no studies performed in LMIC	Byczkowski 2014, Kitayama 2014, O’Connor 2016, Quinlivan 2014, Sharp 2014
**Mothers that shared home-based records with partners or husbands for maternal health increased partners or husbands involvement with pregnancies and helped deal with misconceptions about pregnancy that other family members believed.** Illustrative Quote: Authors stated the MCH handbook helped mothers and caregivers deal with rumours and misconceptions about pregnancy (Hagiwara 2017).	Low confidence	The major concerns revolved around the relevance of the finding to the research questions, the limited number of studies, and overall richness of data.	Hagiwara 2013, Phipps 2001, Yanagisawa 2015
**The use of home-based records for child health improved family engagement with child care.** Illustrative Quote: The [record] provided a positive, inviting message to families about being engaged (King 2017).	Low confidence	Moderate concerns about relevance to the research question, major concern about relevance as low-middle income countries not represented	Clendon 2010, Grippo 2008, King 2017
**Home-based records acted as a point of commonality between caregivers/mothers and nurses, and allowed nurses to provide more comprehensive/tailored health education**. Illustrative Quote: “[the book] was like a stepping stone between the both of you” (Clendon 2010).	Low confidence	The major concerns revolved around the relevance of the finding to the research question and limited number of studies.	Hagiwara 2013, Lee 2016, Yanagisawa 2015, Clendon 2010, Hamilton 2012
**The use of home-based records for maternal and child health facilitated continuity of care.** Illustrative Quote: “I think it would help my GP know what the hospitals were doing and stop tests being repeated” (Quinlivan 2014).	Very low confidence	The major concerns revolved around the relevance of the research questions to the finding, setting limitation, limited number of studies and limited number of participants.	Hamilton 2012, Hully 1993, King 2017, Quinlivan 2014
**Healthcare providers value the educational and logistical aspect of home-based records.** Illustrative Quote: Authors stated that nurses valued the child health book because it connected them to families and helped them provide culturally appropriate care (Lee, 2016).	Low confidence	Knowledge did not consistently report about providers valuing the records. Other concern came with the adequacy of the data, in that most studies did not show rich data, saturation or member checking.	Hagiwara 2013, Phipps 2001, Lee 2016, King 2017, Harrison 1998, Grippo 2008
**A study in a low income setting reports that women value the ease, speed and convenience of online home-based records.** **Healthcare practitioners from two low income countries report that they value the design of home-based records.** Illustrative Quote: "You can do a lot of things automatically. It saves a lot of time" (Kitayama, 2014).	Low confidence	Knowledge consistently reported patient and provider values but for different record types. Concern came with the relevance of the finding to the research question, the coherence and the adequacy of the data, in that most studies did not show rich data, saturation or member checking.	Yanagisawa 2015, Kitayama 2014, Harrison 1998

In relation to our research question, these key findings generated the following: Given the widespread use of HBRs across contexts and its impact on knowledge and education, empowerment, and patient-provider interactions, HBRs are acceptable and useful for women, caregivers and healthcare providers. The feasibility of these interventions may vary greatly depending on geographic location, primary care setting in which they are implemented, and design of the record. No studies provided sufficient data on affordability, or focused on low-literacy or nomadic/refugee populations, limiting our ability to make conclusions about equity.

### Acceptability

Evidence from various geographic contexts and different forms of HBRs indicate that women, caregivers and healthcare providers appreciate and value home-based records. Women from high-income countries valued the ease, speed and convenience of online HBRs [12, 27–29]. However, privacy in relation to online medical records was a consistent concern, except for one study that successfully used records as part of a rare disease network [30]. Health care providers in low-income settings value the design of home-based records and preferred them due to their appearance, practical information, convenience and long-term value [31, 32].

### Feasibility, affordability, equity

The qualitative evidence synthesis did not identify findings on feasibility, affordability or equity from the perspectives of mothers, caregivers and healthcare providers.

### Healthcare provider values

Healthcare providers valued the educational and logistical aspect of HBRs, as well as their design [27, 31–36]. In one low-income setting where card-type home-based records were available, healthcare providers preferred integrated handbooks in terms of its appearance, information, convenience and long-term value [32]. Clinical staff noted the importance of stakeholder engagement in card design to ensure its acceptability and use in primary care settings [31].

### Mother, caregiver and provider interactions

HBRs facilitated communication between mothers/caregivers and health care professionals and improved person-centered care [12, 29, 30, 33–42]. Pregnant women and parents noted decreased fear and improved sense of empowerment during patient–provider interactions [12, 29, 35, 37–39, 41, 42]. HBRs also acted as a point of commonality between caregivers/mothers and nurses and allowed nurses to provide more comprehensive and tailored health education [32, 33, 35, 37, 39]. HBRs have the potential to foster closer relationships between mothers and their healthcare providers [37, 38].

### Improved knowledge and decision making

Increased knowledge emerged as a key finding among pregnant women and caregivers. Parents agreed that they were better able to understand their child's health status, and pregnant women felt that their increased knowledge helped them share in decision-making [27, 30, 32, 34, 35, 42, 43]. However, in one study [42], these views were expressed specifically towards the inclusion of a birth plan within a HBR, and not to the HBR as a whole.

### Communication within the household

HBRs provided a mechanism for increasing husbands’ involvement with pregnancy and address other family members’ misconceptions about pregnancy [32–34]. HBRs similarly provided a mechanism for engaging family with childcare [36–38]. For example, HBRs provided opportunities for women to share information with husbands, partners, and grandparents [34]. In low-literacy settings, some husbands explained the contents of the handbook to their wives and advised them to obtain ANC, avoid salty food, or refrain from working too hard [32]. Among some families, the HBRs represented an intergenerational tool that could be passed down from mother to daughter as she transitioned to motherhood [37].

### Continuity of care

Finally, the use of HBRs for maternal and child health facilitated the continuity of care [29, 36, 39, 41] and facilitated a child’s transition to the adult healthcare system [36].

## Discussion

The UN Sustainable Development Goals called for the adoption and strengthening of sound policies that promote gender equality, the empowerment of all women and improvements in maternal and child health [4]. The WHO is responsible for providing guidance on interventions that have the potential to improve outcomes in both health and empowerment at the primary-care level. The findings of this review confirm that women, caregivers and providers from a wide range of cultural and social contexts engage positively with HBRs.

Within our review we identified ten key findings, across individual, interpersonal, social and organizational levels, which showed connections operating at these levels with the core competencies of community primary care. The majority of our key findings were of low confidence, indicating that the findings may be a reasonable representation of our phenomena of interest. HBRs were valued for improving health knowledge and facilitating women’s communication with health care providers. Knowledge can bring power and vision to disadvantaged communities [44]. The lack of basic health literacy often limits interpersonal communication during health care visits. Improved communication can facilitate intervention outcomes in person-centred care and improve the satisfaction and continuity of care [45]. Continuity between patients and their providers or clinics is a core principle for primary care and an important determinant of the effectiveness of intervention [46].

Home-based records may give mothers and other caregivers a feeling of control and empowerment during clinic visits. Empowerment can improve health and social outcomes, when interventions are embedded in local contexts and are based on strong and direct relationships between people and their health providers [46]. In our review, we found that as mothers feel more in control, they also report feeling less fear during patient–provider interactions. This decrease in fear may lead to fewer barriers to health care access, more opportunities to ask questions, ensure follow-up visits, and help patients develop relationships with their health care providers. A well-maintained home-based record may provide a good first impression, reflect positively on the mother, and be well-perceived by a nurse [47]. While primary care does mean the provision of acute care, the relationships established, the preventive interventions and the improvements in health literacy that come from regular visits provide communities with the most effective care [46, 48].

Clinic staff support the concept of the HBR, but they do not always support its composition [31]. A clinic may face a range of record formats and training may be limited. Also, there is a lack of coordination between the different units of health systems and this leads to reduced use of the HBR [40]. Since health providers value the educational and logistical aspect of home-based records, for the records to be able to meet their needs, it is important that HBRs be designed and implemented with their input [31, 33–36, 38]. It is also vital for health providers, at different levels, to be trained on the use of HBRs. Nurses in low- and middle-income countries and caregivers from low-income populations in the US noted that children’s home-based records should be in the parents' home language and be free of medical jargon [27, 31]. There may be challenges in aligning HBRs with their feasibility at the country level [49]. HBRs, alone, do not lead to behavioural changes in, for example, smoking cessation, drinking alcohol or breastfeeding, without being linked to robust support programs. To ensure results, these elements may require programs in behaviour change [50]. Different levels of the social-ecological framework influence the feasibility, acceptability and use of the home-based record in different contexts. Individuals have their own characteristics and beliefs, but they may also be influenced by family practices and traditions. The engagement of men in pregnancies increases family-level involvement, and HBRs foster a sense of community and relationships between nurses and parents. At the health-system level, public clinics may be more likely to use these records than private clinics.

Health inequities, including barriers to healthcare, are a global challenge for many women and children, worldwide [51]. In different healthcare settings, many women struggle with low literacy and may feel disempowered in their relationships with health providers and in society [51]. When an intervention, such as a home-based record, is available for the entire population, this has implications for positive health equity and also presents opportunities [52]. When a home-based record provides new knowledge, and this new knowledge leads to improved communication, empowerment and continuity of care, we begin to see its importance to and potential for health equity. Ensuring HBRs are written at an appropriate literacy level will help foster this potential.

With the emergence of electronic records, some may argue that these technologies may be the future of health care [53]. This would depend on the scalability of this intervention in low- and middle-income countries, the availability of infrastructure, and individuals’ trust in online records. Mothers reported privacy concerns in relation to online records [12, 27–29]. However, in one study, parents had minimal concerns about confidentiality of online medical records [30]. Trust may vary in this study because the intervention is meant for a specialty based population—children with rare chronic disease in the US. Overall, in all populations, online records appear to offer opportunities for knowledge and engagement. For example, low-income Latina mothers indicated the usefulness of online immunisation records because they remove barriers to accessing and sharing health information [27]. With this increase in knowledge, they also reported wanting to gain more knowledge on the specific immunisations their children were receiving [27]. While the use of online records seems to be acceptable among low-income populations in high-income countries, there is a lack of evidence on their use in low- and middle-income countries. However, the adoption of the electronic health record would appear feasible, based on the widespread use of smartphones among low-income populations [12]. Nevertheless, there is concern about privacy and security; there is also a risk of harm to health equity when certain populations cannot take advantage of new technology.

### Limitations

This review used secondary data, and as such is limited by the information provided in the published primary studies. Several studies included only basic qualitative data and did not provide clear evidence of saturation or data richness. Consequently, details around the core findings, the usability of the HBR and the depth of its community impact are less confident. Only four studies were conducted in low- or middle-income countries, limiting the generalizability of findings to resource-limited or fragile health system contexts. Finally, HBRs have emerged in many different cultural, linguistic and health-education formats, and this heterogeneity made it difficult to provide specific evaluations of HBR usability across regions.

### Strengths

This review utilizes the social-ecological model as a framework for analysis and the GRADE CERQual approach to synthesize the qualitative findings and new understanding of the impact the home-based maternal and child health record has on knowledge, communication skills, and empowerment. The findings also inform the general principles behind the maternal, immunisation and child-health records, providing some basic insight into how and when these records may work and when they may not. The findings of this review also complement the concurrent review of effectiveness of HBRs on maternal newborn and child health outcomes [1], where HBRs were demonstrated to improve knowledge outcomes, communication, and agency. Unique to this review, HBRs increased husbands’ involvement with pregnancies and helped deal with misconceptions that other family members have about pregnancy. Combining this qualitative understanding with quantitative evidence collected to inform WHO recommendations offers a compelling body of knowledge on home-based records.

## Conclusions

The experience of women, caregivers and providers clearly illustrates how HBRs can empower women and children. Women across countries spoke of improved maternal health, communication, and patient centeredness. Women living with low literacy and those in areas with less-developed health care systems reported positive interactions and care continuity. In general, women reported obtaining more learning from nurses and support during pregnancy, with decreased fear and increased empowerment, when HBRs were used. In general, frontline nurses confirmed the acceptability and value added of home-based records. Mothers who used online records had concerns about privacy; however, similar data on patients’ perceptions of online records is scarce and more research is needed. Policy makers need to take stakeholder’s perceptions on the value of home-based records into consideration when making decisions on the use of home-based records in their context.

## Supporting information

S1 Table Example search strategy. (PDF)

S2 Table PICO inclusion and exclusion criteria. (PDF)

S3 Table CERQual evidence profile. (PDF)

S4 Table Table of excluded studies. (PDF)

S1 File WHO qualitative review protocol. (PDF)

S2 File PRISMA checklist. (DOC)
