# Supplementary material for: Understanding women’s, caregivers’, and providers’ experiences with home-based records: A systematic review of qualitative studies
Source: PLoS One. 2018 Oct 4;13(10):e0204966. doi: 10.1371/journal.pone.0204966 (PMC6171900; doi:10.1371/journal.pone.0204966)
Supplement: S1 Table — (PDF) [file pone.0204966.s001.pdf]

Appendix 1: Example Search Strategy

Database: Ovid MEDLINE(R) 1946 to Present with Daily Update, Ovid MEDLINE(R) Epub Ahead of Print <July 31, 2017>, Ovid MEDLINE(R) In-Process & Other Non-Indexed Citations <July 31, 2017>, Ovid MEDLINE(R) Versions
Search Date: 1 August 2017

----

1 exp pregnancy/ (841305)
2 pregnant women/ (6362)
3 pregnan$.tw,kw. (468321)
4 prenatal care/ (24281)
5 perinatal care/ (3796)
6 postnatal care/ (4878)
7 (antenatal or perinatal or postnatal or postpartum or prenatal).mp. (341610)
8 self care/ (30419)
9 mothers/ (35953)
10 (maternal or mother$).mp. (406101)
11 exp child/ (1773552)
12 child$.mp. (2226827)
13 exp infant/ (1067470)
14 (infanc$ or infant? or neonat$ or newborn?).mp. (1360939)
15 or/1-14 (3661435)
16 exp medical records/ (133183)
17 "forms and records control"/ (7928)
18 ((health or medical) adj2 (booklet? or record?)).mp. (183964)
19 ((home-based or patient-held) adj5 (booklet? or handbook? or record?)).tw,kw. (135)
20 ((antenatal or child$) adj3 (booklet? or handbook? or record?)).tw,kw. (5924)
21 ((emr or hbmr or mch or phr) adj5 (booklet? or handbook? or record?)).tw,kw. (1950)
22 ((immunis$ or immuniz$ or vaccinat$) adj2 (booklet? or card? or record?)).tw,kw. (1270)
23 or/16-22 (224969)
24 nurse-patient relations/ (34179)
25 health knowledge, attitudes, practice/ (94833)
26 patient participation/ (22433)
27 patient satisfaction/ (72784)
28 patient preference/ (5808)
29 ((acceptable or acceptabilit$ or complet$ or impact or involved or involvement or participat$ or prefer$ or retain$ or retention or satisf$ or useful$ or utility or value$) adj6 (mother$ or patient?)).tw,kw. (544665)
30 health services accessibility/ (64198)
31 ((access or accessib$) adj5 (care or health$)).tw,kw. (48006)
32 health equity/ (286)
33 ((affordable or affordabilit$ or equitab$ or equity) adj5 (care or health$ or record?)).tw,kw. (12174)
34 (evaluat$ adj5 (care or health$ or record?)).tw,kw. (73223)
35 or/24-34 (876581)
36 15 and 23 and 35 (5095)
37 animals/ not (humans/ and animals/) (4412643)
38 36 not 37 (5090)
39 (1992$ or 1993$ or 1994$ or 1995$ or 1996$ or 1997$ or 1998$ or 1999$ or 2000$ or 2001$ or 2002$ or 2003$ or 2004$ or 2005$ or 2006$ or 2007$ or 2008$ or 2009$ or 2010$ or

2011$ or 2012$ or 2013$ or 2014$ or 2015$ or 2016$ or 2017$).dc,dp,ed,ep,yr. and 38 (4820)
40 remove duplicates from 39 (4578)
