# Supplementary material for: Understanding women’s, caregivers’, and providers’ experiences with home-based records: A systematic review of qualitative studies
Source: PLoS One. 2018 Oct 4;13(10):e0204966. doi: 10.1371/journal.pone.0204966 (PMC6171900; doi:10.1371/journal.pone.0204966)
Supplement: S2 Table — (PDF) [file pone.0204966.s002.pdf]

**Appendix II: PICO Inclusion and Exclusion criteria**

1. **Study design**: The study under consideration must be either a qualitative or mixed method study (with a qualitative component). To be considered for inclusion, a qualitative method must have been used, such as interviews, open-ended surveys, focus groups, or oral history. Additionally, the results must have been analysed and reported qualitatively, such as by using a thematic or framework analysis. Studies which only analyse and report outcomes quantitatively will be excluded.

2. **Population:** The study participants must be related to MNCH. This includes: pregnant women, mothers, infants and children, fathers, caregivers. Individuals other than mothers/fathers may be considered as caregivers if they are the primary individual caring for a child (ex: grandparents, other family relative).

3. **Intervention:** The study under consideration must comment on the use of home-based records. This includes: vaccination only records (record of basic identifying information and immunization services received), vaccination-plus records (record of child growth and development, immunization services, and a limited set of basic information related to child survival), child health book (record of birth characteristics, health services received, growth and feeding practices, guidance to parents), pregnancy case-notes, and maternity personal health records (PHRs). These health records may be in paper form or electronic form to be considered for this review. Patient diaries will not be considered as an eligible intervention.

4. **Outcomes**: Findings reported in the study must related to the research question: Are either single or multi domain home-based records feasible, acceptable, affordable and equitable from the perspective of women, family members, and health providers? Studies which do not offer rich qualitative data related to feasibility, acceptability, affordability or equity will not be considered.

5. **Date:** Eligible studies will be restricted to dates of publication from 1992 – 2017.
