# Supplementary material for: Understanding women’s, caregivers’, and providers’ experiences with home-based records: A systematic review of qualitative studies
Source: PLoS One. 2018 Oct 4;13(10):e0204966. doi: 10.1371/journal.pone.0204966 (PMC6171900; doi:10.1371/journal.pone.0204966)
Supplement: S3 Table — (PDF) [file pone.0204966.s003.pdf]

CERQual Evidence Profile

Key Finding	Studies contributing to the review finding	Assessment of Methodological Limitations	Assessment of Relevance - Research Question	Assessment of Relevance - LMIC or high-income setting	Assessment of Coherence	Assessment of Adequacy of Data	Overall CERQual Assessment of Confidence	Explanation of Judgement
Illustrative Quote: "Love the fact that [the child health record] kept me informed about my child's health" (Kelly, 2017).<br><br>**Home-based records improve the knowledge of mothers and help them share in pregnancy decision making, and improve caregiver's knowledge about their child's health status.**	Phipps 2001, Yanagisawa 2015, Byczkowski 2014, Kelly 2017, Lee 2016, Kitayama 2014, Whitford 2014	**Moderate methodological limitations.**<br><br>Average CASP rating: 8.7<br><br>Justification of qualitative research methods: there is a limited justification of the research design and analysis process of the studies.	**Moderate concerns about relevance of research question**<br><br>Variance noted in the health record interventions evaluated. Some were older, some longer, and some more related to health promotion. In one study (Whitford 2014), mothers expressed views on the inclusion of a birth plan in the home-based record, and not on the record itself.	**Minor concerns about setting relevance**<br><br>Low to high income settings around the world. The 7 studies is not sufficient to study all setting variations.	**Minor concerns about coherence**<br><br>MCHR increases knowledge among users. Results report similar findings and are supported by qualitative quotations.	**Moderate concerns about adequacy**<br><br>Due to limited richness and quantity of data and participants.	**Low Confidence**	Knowledge consistently reported benefit for records even across a range of record style. The major concerns came with the adequacy of the data, in that many studies did not show rich data, saturation or member checking.
Illustrative Quote: "I found the book worked really well, that it was like a communication between the both of you" (Clendon 2010).<br><br>**The use of home-based records for maternal and child health facilitated communication between mothers/caregivers and healthcare professionals and improved person-centered care**.	Byczkowski 2014, Clendon 2010, Grippo 2008, Hagiwara, 2013, Hamilton 2012, Hunter 2008, Hully 1993, Lee 2016, King 2017, Phipps 2001, Quinlivan 2014, Sharp 2014	**Moderate methodological limitations.**<br><br>Average CASP rating: 8.8<br><br>Justification of qualitative research methods: there is a limited justification of the research design and analysis process of the studies.	**Moderate concerns about relevance**<br><br>Communication findings were at times not related to the central research question.	**Minor concerns about setting relevance**<br><br>Low to middle to high income settings around the world. The 12 studies is not sufficient to study all setting variations.	**Minor concerns about coherence**<br><br>MCHR increases communication among users. Results report similar findings and are supported by qualitative quotations. There was only one concern regarding the medical jargon but this was amendable to pictures and language changes.	**Moderate concerns about adequacy**<br><br>Due to limited richness and quantity of data and participants.	**Low Confidence**	The major concerns were with the relevance of the findings and their adequacy because of the limited number of participants in studies.

Illustrative Quote: "I think the Passport [health record] opened up a lot of doors" (Lee 2016). "I can control who sees it." (Quinlivan 2014)<br><br>**The use of home-based records for maternal and child health decrease fear among users and improve confidence and feelings of empowerment during patient-provider interactions.**	Clendon 2010, Grippo 2008, Quinlivan 2014, Whitford 2014, Hamilton 2012, Hully 1993, Lee 2016, Sharp 2014	**Moderate methodological limitations.**<br><br>Average CASP rating: 8.6<br><br>Justification of qualitative research methods: there is a limited justification of the research design and analysis process of the studies.	**Minor concerns about relevance of research question**<br><br>Variance noted in the health record interventions evaluated. Some were older, some longer, and some more related to health promotion. In one study (Whitford 2014), mother expressed views on the inclusion of a birth plan into the home-based record, and not on the home-based record itself. However, this did not pose sufficient concern over relevance in relation to the values/experiences of records.	**Major concerns for setting coherence**<br><br>Lack of representative of low income countries, although there was middle income Brazil	**Minor concerns about coherence**<br><br>MCHR increases confidence and decreases fear among users. Results report similar findings and are supported by qualitative quotations. There was only one concern about residential care workers reporting that minors in residential homes had a different coloured book than other youth, which stigmatized them. This was amendable by implementing a standard colour to all books.	**Moderate concerns about adequacy**<br><br>Due to limited richness and quantity of data and participants	**Low Confidence**	Across a variety of record types, increase in confidence and decrease in fear were consistently reported. The major concerns revolved around the setting limitation and the overall richness of data.
Illustrative Quote: "I'm not sure I want all my medical information out there to be discovered. [...] I'm not convinced it would be safe." (Quinlivan 2014)<br><br>**Mothers and caregivers had concerns with the privacy of online or electronic health records.**	Byczkowski 2014, Kitayama 2014, O'Connor 2016, Quinlivan 2014, Sharp 2014	**Moderate methodological limitations.**<br><br>Average CASP rating: 8.5<br><br>Justification of qualitative research methods: there is a limited justification of the research design and analysis process of the studies.	**Minor concerns for relevancy of research question**<br><br>All studies aimed to gather parent's perceptions and concerns on the use of electronic records for child health.	**Major concerns for relevancy of settings**<br><br>No studies done in LMICs.	**Major concerns regarding coherence**<br><br>Most studies consistently reported fear of on-line records. One study suggested no major concerns.	**Minor concerns about adequacy**<br><br>Studies provide qualitative findings supported by rich quotations and include large sample sizes.	**Low Confidence**	Fear of privacy reported inconsistently in one study. Relevancy of settings is a concern as no studies performed in LMIC.
Illustrative Quote: Authors stated	Hagiwara 2013,	**Moderate**	**Moderate**	**Minor**	**Minor concerns**	**Moderate**	**Low**	The major concerns

the MCH handbook helped mothers and caregivers deal with rumours and misconceptions about pregnancy (Hagiwara 2017).<br><br>**Mothers that shared home-based records with partners or husbands for maternal health increased partners or husbands involvement with pregnancies and helped deal with misconceptions about pregnancy that other family members believed.**	Phipps 2001, Yanagisawa 2015	**methodological limitations.**<br><br>Average CASP rating: 9<br><br>Justification of qualitative research methods: there is a limited justification of the research design and analysis process of the studies.	**concerns about relevance of research question**<br><br>Research questions varied between parents' perceptions of record and record utility. They did not focus on family members or partners.	**concerns about setting relevance**<br><br>The three studies encompass all setting variations: low, middle and high income settings around the world.	**about coherence**<br><br>MCHR increases partner involvement with pregnancy and helps deal with misconceptions among users and their family members. Results report similar findings.	**concerns about adequacy**<br><br>Due to limited quantity of participants and lack of quotations in studies.	**Confidence**	revolved around the relevance of the finding to the research questions, the limited number of studies, and overall richness of data.
Illustrative Quote: The [record] provided a positive, inviting message to families about being engaged (King 2017).<br><br>**The use of home-based records for child health improved family engagement with child care.**	Clendon 2010, Grippo 2008, King, 2017	**Moderate methodological limitations.**<br><br>Average CASP rating: 8.6<br><br>Justification of qualitative research methods: there is a limited justification of the research design and analysis process of the studies.	**Moderate concerns about relevance of research question**<br><br>Research questions varied between parents' perceptions of records and record utility. They did not focus on family engagement with care.	**Major concerns for setting coherence**<br><br>Lack of representation of low income countries, although there was the middle income country Brazil.	**Minor concerns about coherence**<br><br>Results used child health records and one electronic record.	**Minor concerns about adequacy**<br><br>Signs of in-depth qualitative analysis and good participant sample sizes.	**Low Confidence**	Concerns revolved around limited number of studies, setting limitation and the overall richness of data.
Illustrative Quote: "[the book] was like a stepping stone between the both of you" (Clendon 2010)<br><br>**Home-based records acted as a point of commonality between caregivers/mothers and nurses, and allowed nurses to provide more comprehensive/tailored health education**.	Hagiwara 2013, Lee 2016, Yanagisawa 2015, Clendon 2010, Hamilton 2012	**Moderate methodological limitations.**<br><br>Average CASP rating: 8.8<br><br>Justification of qualitative research methods: there is a limited justification of the research design and analysis	**Moderate concerns about relevance of research question**<br><br>Research questions did not focus on nurse behaviour or their relationship with mothers. Also, variance noted in the health record interventions evaluated: some older, some longer,	**Minor concerns about setting relevance**<br><br>Low to middle to high income settings around the world. May not have sufficient studies to study all setting variations.	**Minor concerns about coherence**<br><br>MCHR increases continuity of care for users. Results report similar findings and are supported by qualitative quotations.	**Moderate concerns about adequacy**<br><br>Due to limited richness and lack of quotations in certain studies.	**Low Confidence**	The major concerns revolved around the relevance of the finding to the research question and limited number of studies.

		process of the studies.	some more related to health promotion.					
Illustrative Quote: "I think it would help my GP know what the hospital were doing and stop tests being repeated" (Quinlivan 2014)<br><br>**The use of home-based records for maternal and child health facilitated continuity of care.**	Hamilton 2012, Hully 1993, King 2017, Quinlivan 2014	**Moderate methodological limitations.**<br><br>Average CASP rating: 8.7<br><br>Justification of qualitative research methods: there is a limited justification of the research design and analysis process of the studies.	**Moderate concerns about relevance of research question**<br><br>Research questions varied between parents' perceptions of record and record utility. They did not focus on continuity of care.	**Major concerns for relevancy of settings**<br><br>No studies done in LMICs.	**Minor concerns about coherence**<br><br>MCHR increases continuity of care for users. Results report similar findings and are supported by qualitative quotations.	**Moderate concerns about adequacy**<br><br>Due to limited quantity of data and participants.	**Very Low Confidence**	The major concerns revolved around the relevance of the research questions to the findings, setting limitations, limited number of studies and limited number of participants.
