# Supplementary material for: Understanding women’s, caregivers’, and providers’ experiences with home-based records: A systematic review of qualitative studies
Source: PLoS One. 2018 Oct 4;13(10):e0204966. doi: 10.1371/journal.pone.0204966 (PMC6171900; doi:10.1371/journal.pone.0204966)
Supplement: S4 Table — (PDF) [file pone.0204966.s004.pdf]

Appendix IV : Table of excluded studies

Citation	Reason for exclusion
Ahlers-Schmidt, C. R., & Nguyen, M. (2013). Parent Intention to Use a Patient Portal as Related to Their Children Following a Facilitated Demonstration. *Telemedicine and E-Health*, *19*(12), 979–981.	Study Design
Aiga, H., Nguyen, V. D., Nguyen, C. D., Nguyen, T. T. T., & Nguyen, L. T. P. (2015). Knowledge, attitude and practices: assessing maternal and child health care handbook intervention in Vietnam. *BMC Public Health*, *16*(1), 129.	Outcomes not relevant to FACE
Anonymous. (2009). Personal health records. Take charge of your health information. *Mayo Clinic Health Letter (English Ed.)*, *27*(2), 7.	Study Design
Archer, N., Fevrier-Thomas, U., Lokker, C., McKibbon, K. A., & Straus, S. E. (2011). Personal health records: a scoping review. *Journal of the American Medical Informatics Association*, *18*(4), 515–522.	Study Design
Atkin, P. A., Finnegan, T. P., Ogle, S. J., & Shenfield, G. M. (1995). Are medication record cards useful? *The Medical Journal of Australia*, *162*(6), 300–1.	Study Design
Atkinson, K. M., Westeinde, J., Ducharme, R., Wilson, S. E., Deeks, S. L., Crowcroft, N., … Wilson, K. (2016). Can mobile technologies improve on-time vaccination? A study piloting maternal use of ImmunizeCA, a Pan-Canadian immunization app. *Human Vaccines & Immunotherapeutics*, *12*(10), 2654–2661.	Study Design
Basaleem, H. O., Al-Sakkaf, K. A., & Shamsuddin, K. (2010). *Saudi Medical Journal. Saudi Medical Journal* (Vol. 31).	Study Design
Bedford, H., & Chalmers, J. (2003). A new vision for maternity records in Scotland: the Scottish Woman-Held Maternity Record (SWHMR) project and the electronic Scottish Woman-Held Maternity Record (eSWHMR) project. *Journal (Institute of Health Record Information and Management)*, *44*(2), 21–4.	Study Design
Benhamou, P.-Y. (2011). Improving diabetes management with electronic health records and patients' health records. *Diabetes & Metabolism*, *37 Suppl 4*, S53-6.	Study Design
Bhargava, A., Guntupalli, A. M., Lokshin, M., & Howard, L. L. (2014). MODELING THE EFFECTS OF IMMUNIZATIONS TIMING ON CHILD HEALTH OUTCOMES IN INDIA. *Health Economics*, *23*(5), 606–620.	Study Design
Billault, B., Degoulet, P., Devries, C., Plouin, P. F., Chatellier, G., & Menard, J. (1995). Use of a standardized personal medical record by patients with hypertension: a randomized controlled prospective trial. *M.D. Computing : Computers in Medical Practice*, *12*(1), 31–5.	Study Design
Bjerkeli Grøvdal, L., Grimsmo, A., & Ivar Lund Nilsen, T. (2006). Parent-held child health records do not improve care: A randomized controlled trial in Norway. *Scandinavian Journal of Primary Health Care*, *24*(3), 186–190.	Study Design

Bouhaddou, O., & Warner, H. (1995). An interactive patient information and education system (Medical HouseCall) based on a physician expert system (Iliad). *Medinfo. MEDINFO*, *8 Pt 2*, 1181–5.	Intervention not related to PICO
Bourgeois, F. C., Taylor, P. L., Emans, S. J., Nigrin, D. J., & Mandl, K. D. (2008). Whose Personal Control? Creating Private, Personally Controlled Health Records for Pediatric and Adolescent Patients. *Journal of the American Medical Informatics Association*, *15*(6), 737–743.	Intervention not related to PICO
Bourgeois, F. C., Mandl, K. D., Shaw, D., Flemming, D., & Nigrin, D. J. (2009). Mychildren's: integration of a personally controlled health record with a tethered patient portal for a pediatric and adolescent population. *AMIA ... Annual Symposium Proceedings. AMIA Symposium*, *2009*, 65–9.	Study Design
Bourke, B., Lyons, D., Clarke, T., & Mathews, T. (1992). Parent held records an evaluation. *Irish Medical Journal*, *85*(3), 118.	Full text could not be retrieved
Burke, R. P., Rossi, A. F., Wilner, B. R., Hannan, R. L., Zabinsky, J. A., & White, J. A. (2010). Transforming patient and family access to medical information: utilisation patterns of a patient-accessible electronic health record. *Cardiology in the Young*, *20*(5), 477–484.	Study Design
Campbell, H., & Halleran, J. (1993). An evaluation of the personal child health record in Fife. *Health Bulletin*, *51*(6), 399–406.	Study Design
Charles, R. (1994). An evaluation of parent-held child health records. *Health Visitor*, *67*(8), 270–2.	Study Design
Chhabra, P., Nair, P., Gupta, A., Sandhir, M., & Kannan, A. T. (2007). Immunization in urbanized villages of Delhi. *The Indian Journal of Pediatrics*, *74*(2), 131–134.	Study Design
Choi, C. S., Brennan, P. F., & Kalish, C. (1998). Child health records: are they valid and useful to children and pediatric practitioners? *Proceedings. AMIA Symposium*, 453–6.	Intervention not related to PICO
Cormack, L., Morley, C., Seward, A., & Vickers, D. (1998). The personal child health record: Attitudes to and usage by parents and professionals during the first year of a child's life. *Ambulatory Child Health*, *4*, 375–380.	Full text could not be retrieved
Council on Clinical Information Technology. (2009). Using Personal Health Records to Improve the Quality of Health Care for Children. *Pediatrics*, *124*(1).	Study Design
Dang, M. T., Whitney, K. D., Virata, M. C. D., Binger, M. M., & Miller, E. (2012). A Web-Based Personal Health Information System for Homeless Youth and Young Adults. *Public Health Nursing*, *29*(4), 313–319.	Intervention not related to PICO
Davies, A. (1999). Parent-held child health records: an evaluation of parental attitudes. *British Journal of Community Nursing*, *4*(5), 242–249.	Study Design
Dearlove, J., & Illingworth, S. (1999). A controlled trial of parent initiated and conventional preschool health surveillance using personal child health records. *Archives of Disease in Childhood*, *80*(6), 507–10.	Study Design

Duclos, V., Yé, M., Moubassira, K., Sanou, H., Sawadogo, N. H., Bibeau, G., & Sié, A. (2017). Situating mobile health: a qualitative study of mHealth expectations in the rural health district of Nouna, Burkina Faso. *Health Research Policy and Systems*, *15*(S1), 47.	Intervention not related to PICO
Esch, T., Mejilla, R., Anselmo, M., Podtschaske, B., Delbanco, T., & Walker, J. (2016). Engaging patients through open notes: an evaluation using mixed methods. *BMJ Open*, *6*(1), e010034.	Intervention not related to PICO
Essén, A., Gerrits, R., & Kuhlmann, E. (2017). Patient accessible electronic health records: Connecting policy and provider action in the Netherlands. *Health Policy and Technology*, *6*(2), 134–141.	Intervention not related to PICO
Fermon, F. (1995). [Lost immunization opportunities in Niamey Niger]. *DEVELOPPEMENT ET SANTE*, (115), 24–7.	Study Design
Fernandez-Luque, L., Karlsen, R., Krogstad, T., Burkow, T. M., & Vognild, L. K. (2010). Personalized health applications in the Web 2.0: The emergence of a new approach. In *2010 Annual International Conference of the IEEE Engineering in Medicine and Biology* (pp. 1053–1056). IEEE.	Study Design
Fernandez, N., Copenhaver, D. J., Vawdrey, D. K., Kotchoubey, H., & Stockwell, M. S. (2017). Smartphone Use Among Postpartum Women and Implications for Personal Health Record Utilization. *Clinical Pediatrics*, *56*(4), 376–381.	Study Design
Fisher, B. (2013). Patients' access to their electronic record: offer patients access as soon as you can. *The British Journal of General Practice : The Journal of the Royal College of General Practitioners*, *63*(611), e423-5.	Study Design
Fisher, B. (2011). Patient record access: making it work for you and the NHS. *London Journal of Primary Care*, *4*(1), 44–49.	Study Design
Fitton, C., Fitton, R., Hannan, A., Fisher, B., Morgan, L., & Halsall, D. (2014). The impact of patient record access on appointments and telephone calls in two English general practices: a population-based study. *London Journal of Primary Care*, *6*(1), 8–15.	Intervention not related to PICO
Forbes, M., Fairlamb, H., & Jonker, L. (2015). The Health Of Patients' Eyes (HOPE) Glaucoma study. The effectiveness of a "glaucoma personal record" for newly diagnosed glaucoma patients: study protocol for a randomised controlled trial. *Trials*, *16*(1), 337.	Study Design
Forster, M., Dennison, K., Callen, J., Georgiou, A., & Westbrook, J. I. (2015). Maternity Patients' Access to Their Electronic Medical Records: Use and Perspectives of a Patient Portal. *Health Information Management Journal*, *44*(1), 4–11.	Study Design
Frampton, S. B., Horowitz, S., & Stumpo, B. J. (2009). Open Medical Records. *AJN, American Journal of Nursing*, *109*(8), 59–63.	Study Design
Frost, J. H., & Massagli, M. P. (2008). Social uses of personal health information within PatientsLikeMe, an online patient community: what can happen when patients have access to one another's data. *Journal of Medical Internet Research*, *10*(3), e15.	Study Design

Fuji, K. T., Abbott, A. A., & Galt, K. A. (2014). Personal health record design: qualitative exploration of issues inhibiting optimal use. *Diabetes Care*, *37*(1), e13-4.	Intervention not related to PICO
Furmanski, L., Bahamonde, L., Cunningham, N., Maw, K. L., Ramos, C., Lobach, K., … Findley, S. E. (1996). A children's lifetime home-based health record: Its use and non-use. *Ambulatory Child Health*, *2*, 139–149.	Full text could not be retrieved
Gaíva, M. A. M., & Silva, F. B. da. (2014). CHILD HEALTH HANDBOOK: INTEGRATIVE REVIEW. *Journal of Nursing UFPE*, *8*(3), 742–749.	Study Design
GEE, P. M., PATERNITI, D. A., WARD, D., & SOEDERBERG MILLER, L. M. (2015). e-Patients Perceptions of Using Personal Health Records for Self-management Support of Chronic Illness. *CIN: Computers, Informatics, Nursing*, *33*(6), 229–237.	Intervention not related to PICO
Gordon, P., Camhi, E., Hesse, R., Odlum, M., Schnall, R., Rodriguez, M., & Valdez, E. (2012). Processes and outcomes of developing a continuity of care document for use as a personal health record by people living with HIV/AIDS in New York City. *International Journal of Medical Informatics*, *81*(10), e63–e73.	Intervention not related to PICO
Griffith, R., & Tengnah, C. (2013). When to allow or deny adult access to children's health records. *British Journal of Community Nursing*, *18*(3), 148–151.	Study Design
Groenen, C. J. M., Faber, M. J., Kremer, J. A. M., Vandenbussche, F. P. H. A., & van Duijnhoven, N. T. L. (2016). Improving maternity care using a personal health record: study protocol for a stepped-wedge, randomised, controlled trial. *Trials*, *17*(1), 202.	Intervention not related to PICO
Hampshire, A. J., Blair, M. E., Crown, N. S., Avery, A. J., & Williams, E. I. (2004). Variation in how mothers, health visitors and general practitioners use the personal child health record. *Child: Care, Health and Development*, *30*(4), 307–316.	Study Design
Harrison, D., Harker, H., Heese, H. V, & Mann, M. (2005). An assessment by nurses and mothers of a "road-to-health " book in the Western Cape. *Curationis*, *28*(4), 57–64.	Study Design
Hassol, A., Walker, J. M., Kidder, D., Rokita, K., Young, D., Pierdon, S., … Ortiz, E. (2004). Patient Experiences and Attitudes about Access to a Patient Electronic Health Care Record and Linked Web Messaging. *Journal of the American Medical Informatics Association*, *11*(6), 505–513.	Intervention not related to PICO
Hennekam, M., Totté, J. E. E., & Pasmans, S. G. M. A. (2014). [E-health: the Skin House]. *Nederlands Tijdschrift Voor Geneeskunde*, *158*, A8394.	Intervention not related to PICO
Hess, R., Bryce, C. L., Paone, S., Fischer, G., McTigue, K. M., Olshansky, E., … Siminerio, L. (2007). Exploring Challenges and Potentials of Personal Health Records in Diabetes Self-Management: Implementation and Initial Assessment. *Telemedicine and E-Health*, *13*(5), 509–518.	Study Design
Hess, R., Fischer, G. S., Sullivan, S. M., Dong, X., Weimer, M., Zeith, C., … Roberts, M. S. (2014). Patterns of Response to Patient-	Study Design

Centered Decision Support Through a Personal Health Record. *Telemedicine and E-Health*, *20*(11), 984–989.	
Holmes, A., Cheyne, H., Ginley, M., & Mathers, A. (2005). Trialling and implementing a client-held record system. *British Journal of Midwifery*, *13*(2), 112–117.	Study Design
Homer, C. S. E., Davis, G. K., & Everitt, L. S. (1999). The Introduction of a Woman-Held Record into a Hospital Antenatal Clinic: The Bring Your Own Records Study. *Australian and New Zealand Journal of Obstetrics and Gynaecology*, *39*(1), 54–57.	Study Design
Hong, M. K., Wilcox, L., Feustel, C., Wasileski-Masker, K., Olson, T. A., & Simoneaux, S. F. (2016). Adolescent and Caregiver use of a Tethered Personal Health Record System. *AMIA ... Annual Symposium Proceedings. AMIA Symposium*, *2016*, 628–637.	Intervention not related to PICO
Hooker, L., & Williams, J. (1996). Parent-held shared care records: bridging the communication gaps. *British Journal of Nursing*, *5*(12), 738–741.	Study Design
Hyne, J. (1999). Personal held records: Encouraging partnership with children and parents. *Paediatric Nursing*, *11*(6), 21–24.	Study Design
JEFFS, D., NOSSAR, V., BAILEY, F., SMITH, W., & CHEY, T. (1994). Retention and use of personal health records: A population-based study. *Journal of Paediatrics and Child Health*, *30*(3), 248–252.	Study Design
Johansen, M. A., & Henriksen, E. (2014). The evolution of personal health records and their role for self-management: a literature review. *Studies in Health Technology and Informatics*, *205*, 458–62.	Study Design
Johnston, D., Kaelber, D., Pan, E. C., Bu, D., Shah, S., Hook, J. M., & Middleton, B. (2007). A framework and approach for assessing the value of personal health records (PHRs). *AMIA ... Annual Symposium Proceedings. AMIA Symposium*, *2007*, 374–8.	Study Design
Jones, A., Henwood, F., & Hart, A. (2002). Electronic patient records: the view from maternity. *British Journal of Midwifery*, *10*(10), 635–639.	Study Design
Jung, E. Y., Park, D. K., Kang, H. W., & Lim, Y. S. (2013). Personalized health management services based on personal health record (PHR). *Studies in Health Technology and Informatics*, *192*, 956.	Study Design
Kawakatsu, Y., Sugishita, T., Oruenjo, K., Wakhule, S., Kibosia, K., Were, E., & Honda, S. (2015). Effectiveness of and factors related to possession of a mother and child health handbook: an analysis using propensity score matching. *Health Education Research*, *30*(6), cyv048.	Study Design
Kendrick, E. J., & Benson, C. (2017). Patient Portals in Child and Adolescent Psychiatry. *Child and Adolescent Psychiatric Clinics of North America*, *26*(1), 43–54.	Intervention not related to PICO
Kilmartin, M. R., Woodward, D. R., Leigh Blizzard, C., & Turner, K. (1998). Immunisation of babies. The mothers' perspective. *Australian Family Physician*, *27 Suppl 1*, S11-4.	Intervention not related to PICO
Kim, H.-K., Nam, J.-E., Chang, W.-Y., Rho, Y.-K., & Choi, M.-K. (2012). Retention of the Mother and Child Health Handbook and	Intervention is not focused on Handbook

Additional Immunization of Japanese Encephalitis and Tetanus Vaccine. *Korean Journal of Family Medicine*, *33*(4), 237.	
Kim, M. I., & Johnson, K. B. (2002). Personal Health Records: Evaluation of Functionality and Utility. *Journal of the American Medical Informatics Association*, *9*(2), 171–180.	Study Design
King, G., Maxwell, J., Karmali, A., Hagens, S., Pinto, M., Williams, L., & Adamson, K. (2017). Connecting Families to Their Health Record and Care Team: The Use, Utility, and Impact of a Client/Family Health Portal at a Children's Rehabilitation Hospital. *Journal of Medical Internet Research*, *19*(4), e97.	Intervention not related to PICO
Kiran, T. S. U., & Jayawickrama, N. S. (2002). Hand-held maternity records: are they an added burden? *Journal of Evaluation in Clinical Practice*, *8*(3), 349–352.	Study Design
Kruse, C. S., Argueta, D. A., Lopez, L., & Nair, A. (2015). Patient and provider attitudes toward the use of patient portals for the management of chronic disease: a systematic review. *Journal of Medical Internet Research*, *17*(2), e40.	Study Design
Kumar, V., & Datta, N. (1988). Home-based mothers' health records. *World Health Forum*, *9*(1), 107–10.	Study Design
Lakhani, A. D., Avery, A., Gordon, A., & Tait, N. (1984). Evaluation of a home based health record booklet. *Archives of Disease in Childhood*, *59*(11), 1076–81.	Study Design
Lau, A. Y., Sintchenko, V., Crimmins, J., Magrabi, F., Gallego, B., & Coiera, E. (2012). Protocol for a randomised controlled trial examining the impact of a web-based personally controlled health management system on the uptake of influenza vaccination rates. *BMC Health Services Research*, *12*(1), 86.	Study Design
Lau, M., Campbell, H., Tang, T., Thompson, D. J. S., & Elliott, T. (2014). Impact of Patient Use of an Online Patient Portal on Diabetes Outcomes. *Canadian Journal of Diabetes*, *38*(1), 17–21.	Study Design
Leodolter, I. (1978). Short report: The mother-child health passport: Austria's successful weapon against infant mortality. *Preventive Medicine*, *7*(4), 561–563.	Study Design
Liaw, S. T., Radford, A. J., & Maddocks, I. (1998). The impact of a computer generated patient held health record. *Australian Family Physician*, *27 Suppl 1*, S39-43.	Study Design
Lovell, A., Zander, L. I., James, C. E., Foot, S., Swan, A. V., & Reynolds, A. (1987). The St. Thomas's Hospital maternity case notes study: A randomised controlled. trial to assess the effects of giving expectant mothers their own maternity case notes. *Paediatric and Perinatal Epidemiology*, *1*(1), 57–66.	Study Design
Luque, A. E., Corales, R., Fowler, R. J., DiMarco, J., van Keken, A., Winters, P., … Fiscella, K. (2013). Bridging the Digital Divide in HIV Care. *Journal of the International Association of Providers of AIDS Care (JIAPAC)*, *12*(2), 117–121.	Study Design
Lyles, C. R., Harris, L. T., Le, T., Flowers, J., Tufano, J., Britt, D., … Ralston, J. D. (2011). Qualitative Evaluation of a Mobile Phone and Web-Based Collaborative Care Intervention for Patients	Intervention not related to PICO

with Type 2 Diabetes. *Diabetes Technology & Therapeutics*, *13*(5), 563–569.	
Matsuda, Y., Manaka, T., Kobayashi, M., Sato, S., & Ohwada, M. (2016). Exploratory analysis of textual data from the *Mother and Child Handbook* using the text-mining method: Relationships with maternal traits and post-partum depression. *Journal of Obstetrics and Gynaecology Research*, *42*(6), 655–660.	Study Design
McCormick, M. C., Shapiro, S., & Starfield, B. H. (1981). The Association of Patient-Held Records and Completion of Immunizations. *Clinical Pediatrics*, *20*(4), 270–274.	Study Design
Mickelson, R. S., Willis, M., & Holden, R. J. (2015). Medication-related cognitive artifacts used by older adults with heart failure. *Health Policy and Technology*, *4*(4), 387–398.	Study Design
Mold, F., de Lusignan, S., Sheikh, A., Majeed, A., Wyatt, J. C., Quinn, T., … Ellis, B. (2015). Patients' online access to their electronic health records and linked online services: a systematic review in primary care. *The British Journal of General Practice : The Journal of the Royal College of General Practitioners*, *65*(632), e141-51.	Study Design
Mora, F. (2012). The demise of Google Health and the future of personal health records. *International Journal of Healthcare Technology and Management*, *13*(5/6), 363.	Study Design
Moss, A. L. H. (2005). Is the personal child health record used in secondary care? *Child: Care, Health and Development*, *31*(5), 627–628.	Study Design
Mudany, M. A., Sirengo, M., Rutherford, G. W., Mwangi, M., Nganga, L. W., & Gichangi, A. (2015). Enhancing Maternal and Child Health using a Combined Mother & Child Health Booklet in Kenya. *Journal of Tropical Pediatrics*, *61*(6), fmv055.	Outcomes not relevant to FACE
Mukanga, D. O., & Kiguli, S. (2006). Factors Affecting the Retention and Use of Child Health Cards in a Slum Community in Kampala, Uganda, 2005. *Maternal and Child Health Journal*, *10*(6), 545–552.	Study Design
Naik, A. D., Schulman-Green, D., McCorkle, R., Bradley, E. H., & Bogardus, S. T. (2005). Will Older Persons and Their Clinicians Use a Shared Decision-making Instrument? *Journal of General Internal Medicine*, *20*(7), 640–643.	Intervention not related to PICO
Nash, L., Dixon, R., Eaton, V., & Grzeskowiak, L. E. (2015). Accuracy of information on medication use and adverse drug reactions recorded in pregnancy hand-held records. *Australian and New Zealand Journal of Obstetrics and Gynaecology*, *55*(6), 547–551.	Study Design
Nuti, L., Turkcan, A., Lawley, M. A., Zhang, L., Sands, L., & McComb, S. (2015). The impact of interventions on appointment and clinical outcomes for individuals with diabetes: a systematic review. *BMC Health Services Research*, *15*, 355.	Study Design
O'Flaherty, S., Jandera, E., Llewellyn, J., & Wall, M. (1987). Personal health records: an evaluation. *Archives of Disease in Childhood*, *62*(11), 1152–5.	Study Design

Or, C. K. L., Karsh, B.-T., Severtson, D. J., Burke, L. J., Brown, R. L., & Brennan, P. F. (2011). Factors affecting home care patients' acceptance of a web-based interactive self-management technology. *Journal of the American Medical Informatics Association*, *18*(1), 51–59.	Study Design
Osaki, K., Hattori, T., Kosen, S., & Singgih, B. (2009). Investment in home-based maternal, newborn and child health records improves immunization coverage in Indonesia. *Transactions of the Royal Society of Tropical Medicine and Hygiene*, *103*(8), 846–848.	Study Design
Pahari, D. P., Bastola, S. P., & Paudel, R. (2011). Factors Affecting Retention of Child Health Card in a Rural Area. *Journal of Nepal Health Research Council*, *9*(19).	Study Design
Palombo, C. N. T., Duarte, L. S., Fujimori, E., Toriyama, Á. T. M., Palombo, C. N. T., Duarte, L. S., … Toriyama, Á. T. M. (2014). Use and records of child health handbook focused on growth and development. *Revista Da Escola de Enfermagem Da USP*, *48*(spe), 59–66.	Study Design
Patterson, K., & Logan-Sinclair, P. (2003). Continuum of care and the antenatal record in rural New South Wales. *Australian Journal of Rural Health*, *11*(3), 110–115.	Intervention not related to PICO
Phipps, H. (2001). Carrying their own medical records: the perspective of pregnant women. *The Australian and New Zealand Journal of Obstetrics and Gynaecology*, *41*(4), 398–401.	Intervention not related to PICO
Popovich, M. L., Aramini, J. J., & Garcia, M. (2008). Immunizations: the first step in a personal health record to empower patients. *Studies in Health Technology and Informatics*, *137*, 286–95.	Study Design
Powell, J., Fitton, R., & Fitton, C. (2006). Sharing electronic health records: the patient view. *Journal of Innovation in Health Informatics*, *14*(1), 55–57.	Study Design
Prashad, R. (2017). The Role of Personal Health Record Systems in Chronic Disease Management. *Studies in Health Technology and Informatics*, *234*, 275–279.	Study Design
Pushpangadan, S., & Seckman, C. (2015). Consumer Perspective on Personal Health Records: A Review of the Literature. *Online Journal of Nursing Informatics*, *19*(1), 2.	Study Design
Requejo, J. H., Merialdi, M., & Bustreo, F. (2011). Improving global maternal health. *Current Opinion in Obstetrics and Gynecology*, *23*(6), 465–470.	Study Design
Riippa, I., Linna, M., & Rönkkö, I. (2014). The effect of a patient portal with electronic messaging on patient activation among chronically ill patients: controlled before-and-after study. *Journal of Medical Internet Research*, *16*(11), e257.	Study Design
Ronis, S. D., Baldwin, C. D., McIntosh, S., McConnochie, K., Szilagyi, P. G., & Dolan, J. (2015). Caregiver Preferences Regarding Personal Health Records in the Management of ADHD. *Clinical Pediatrics*, *54*(8), 765–774.	Study Design
Santos Neto, E. T., Oliveira, A. E., Zandonade, E., Gama, S. G., & Leal Mdo, C. (2012). [Prenatal patient cards and quality of prenatal	Study Design

care in public health services in Greater Metropolitan Vitoria, Espirito Santo State, Brazil]. *Cadernos de Saude Publica*, *28*, 1650–1662.	
Spooner, S. A., & Council on Clinical Information Technology, American Academy of Pediatrics. (2007). Special requirements of electronic health record systems in pediatrics. *Pediatrics*, *119*(3), 631–7.	Study Design
Schoevers, M. A., van den Muijsenbergh, M. E. T. C., & Lagro-Janssen, A. L. M. (2009). Patient-held records for undocumented immigrants: a blind spot. A systematic review of patient-held records. *Ethnicity & Health*, *14*(5), 497–508.	Intervention not related to PICO
Scott, S., & Illingworth, S. (1998). Intensive interventions to improve parenting. *Archives of Disease in Childhood*, *79*(1), 90–3.	Intervention not related to PICO
SENANAYAKE, I. P. (1977). Use of Home Based Records in the Evaluation of a Health Care System. *Journal of Tropical Pediatrics*, *23*(5), 220–223.	Year outside of accepted range
Shah, P. M., Selwyn, B. J., Shah, K., & Kumar, V. (1993). Evaluation of the home-based maternal record: a WHO collaborative study. *Bulletin of the World Health Organization*, *71*(5), 535–48.	Study Design
Shaw, E., Howard, M., Chan, D., Waters, H., Kaczorowski, J., Price, D., & Zazulak, J. (2008). Access to Web-Based Personalized Antenatal Health Records for Pregnant Women: A Randomized Controlled Trial. *Journal of Obstetrics and Gynaecology Canada*, *30*(1), 38–43.	Study Design
SHAW, R. J., & FERRANTI, J. (2011). Patient-Provider Internet Portals—Patient Outcomes and Use. *CIN: Computers, Informatics, Nursing*, *29*(12), 714–718.	Study Design
Simba, D. (2009). Towards a sustainable community database: taking advantage of the Road-to-Health cards to monitor and evaluate health interventions targeting under fives. *Tanzania Journal of Health Research*, *11*(1).	Study Design
Somner, J. E., Sii, F., Bourne, R., Cross, V., & Shah, P. (2013). What do patients with glaucoma think about personal health records? *Ophthalmic and Physiological Optics*, *33*(6), 627–633.	Intervention not related to PICO
Sox, C. M., Gribbons, W. M., Loring, B. A., Mandl, K. D., Batista, R., & Porter, S. C. (2010). Patient-centered design of an information management module for a personally controlled health record. *Journal of Medical Internet Research*, *12*(3), e36.	Intervention not related to PICO
Stacy, R. D., Sharma, M., & Torrence, W. A. (2008). Evaluation of the Use of a Parent-Held Child Health Record by Pregnant Women and Mothers of Young Children. *Californian Journal of Health Promotion*, *6*(1), 138–142.	Study Design
Stephens, B., Gowers, P., & Kennedy, I. (1977). Patient retained health records in a rural health care system. *The Journal of Tropical Medicine and Hygiene*, *80*(11), 244–8.	Year outside of accepted range
Tang, P. C., Ash, J. S., Bates, D. W., Overhage, J. M., & Sands, D. Z. (2006). Personal Health Records: Definitions, Benefits, and Strategies for Overcoming Barriers to Adoption. *Journal of the American Medical Informatics Association*, *13*(2), 121–126.	Study Design

Tenforde, M., Jain, A., & Hickner, J. (2011). The value of personal health records for chronic disease management: what do we know? *Family Medicine*, *43*(5), 351–4.	Study Design
Tenforde, M., Nowacki, A., Jain, A., & Hickner, J. (2012). The Association Between Personal Health Record Use and Diabetes Quality Measures. *Journal of General Internal Medicine*, *27*(4), 420–424.	Study Design
Tom, J. O., Chen, C., & Zhou, Y. Y. (2014). Personal health record use and association with immunizations and well-child care visits recommendations. *The Journal of Pediatrics*, *164*(1), 112–7.	Study Design
Tom, J. O., Mangione-Smith, R., Solomon, C., & Grossman, D. C. (2012). Integrated personal health record use: association with parent-reported care experiences. *Pediatrics*, *130*(1), e183-90.	Study Design
Toohill, J., Soong, B., & Meldrum, M. (2006). Risk management considerations and the pregnancy handheld record. *Women and Birth*, *19*(4), 113–116.	Study Design
Troude, P., L'Hélias, L. F., Raison-Boulley, A.-M., Castel, C., Pichon, C., Bouyer, J., & de La Rochebrochard, E. (2008). Perinatal factors reported by mothers: do they agree with medical records? *European Journal of Epidemiology*, *23*(8), 557–564.	Study Design
Turner, K., Klaman, S. L., & Shea, C. M. (2016). Personal health records for people living with HIV: a review. *AIDS Care*, *28*(9), 1181–1187.	Study Design
Usman, H. R., Akhtar, S., Habib, F., & Jehan, I. (2009). Redesigned immunization card and center-based education to reduce childhood immunization dropouts in urban Pakistan: A randomized controlled trial. *Vaccine*, *27*(3), 467–472.	Study Design
Usman, H. R., Rahbar, M. H., Kristensen, S., Vermund, S. H., Kirby, R. S., Habib, F., & Chamot, E. (2011). Randomized controlled trial to improve childhood immunization adherence in rural Pakistan: redesigned immunization card and maternal education. *Tropical Medicine & International Health*, *16*(3), 334–342.	Study Design
van der Hoek, W., Ngoma, M., & Soeters, R. (1994). The Home-Based Health Passport: A Tool for Primary Health Care. *Tropical Doctor*, *24*(1), 21–23.	Study Design
Vermeir, P., Degroote, S., Vandijck, D., Van Tiggelen, H., Peleman, R., Verhaeghe, R., … Vogelaers, D. (2017). The patient perspective on the effects of medical record accessibility: a systematic review. *Acta Clinica Belgica*, *72*(3), 186–194.	Study Design
Vincelet, C., Tabone, M. D., Berthier, M., Bonnefoi, M. C., Chevallier, B., Lemaire, J. P., … Groupe de pédiatrie générale de la Société française de pédiatrie. (2003). [How are personal child health records completed? A multicentric evaluation study]. *Archives de Pediatrie : Organe Officiel de La Societe Francaise de Pediatrie*, *10*(5), 403–9.	Study Design
VOLKMER, R. E., GOULDSTONE, M. A., & NINNES, C. P. (2008). Parental perception of the use and usefulness of a parent-held	Study Design

child health record. *Journal of Paediatrics and Child Health*, *29*(2), 150–153.	
Wäckerle, A., Blöchlinger-Wegmann, B., Burkhardt, T., Krähenmann, F., Kurmanavicius, J., & Zimmermann, R. (2010). Notes on a stick: use and acceptability of woman-held maternity notes. *European Journal of Obstetrics & Gynecology and Reproductive Biology*, *153*(2), 156–159.	Study Design
Wagner, P. J., Dias, J., Howard, S., Kintziger, K. W., Hudson, M. F., Seol, Y.-H., & Sodomka, P. (2012). Personal health records and hypertension control: a randomized trial. *Journal of the American Medical Informatics Association*, *19*(4), 626–634.	Study Design
Walton, S., & Bedford, H. (2007). Parents' use and views of the national standard Personal Child Health Record: a survey in two primary care trusts. *Child: Care, Health and Development*, *33*(6), 744–748.	Study Design
Webster, J., Forbes, K., Foster, S., Thomas, I., Griffin, A., & Timms, H. (1996). Sharing Antenatal Care: Client Satisfaction and Use of the "Patient-held Record." *The Australian and New Zealand Journal of Obstetrics and Gynaecology*, *36*(1), 11–14.	Study Design
Weinert, C., & Cudney, S. (2012). My Health Companion©: A Low-Tech Personal Health Record Can Be an Essential Tool for Maintaining Health. *Online Journal of Rural Nursing and Health Care*, *12*(1), 3–15.	Intervention not related to PICO
Weithase, A., Vogel, M., Kiep, H., Schwarz, S., Meißner, L., Herrmann, J., … Kiess, W. (2017). [Quality of and Attendance at Healthy Child Clinics in Germany]. *Deutsche Medizinische Wochenschrift (1946)*, *142*(8), e42–e50.	Study Design
Weitzman, E. R., Kaci, L., & Mandl, K. D. (2009). Acceptability of a personally controlled health record in a community-based setting: implications for policy and design. *Journal of Medical Internet Research*, *11*(2), e14.	Intervention not related to PICO
Williams, B. T., Imrey, H., & Williams, R. G. (1991). The lifespan personal health record. *Medical Decision Making : An International Journal of the Society for Medical Decision Making*, *11*(4 Suppl), S74-6.	Study Design
Wilson, K., Atkinson, K., Pluscauskas, M., & Bell, C. (2014). A mobile-phone immunization record in Ontario: uptake and opportunities for improving public health. *Journal of Telemedicine and Telecare*, *20*(8), 476–480.	Study Design
Wright, C. M., & Reynolds, L. (2006). How widely are personal child health records used and are they effective health education tools? A comparison of two records. *Child: Care, Health and Development*, *32*(1), 55–61.	Study Design
Zimmermann, R., Blochlinger-Wegmann, B., & Kurmanavicius, J. (2006). The electronic maternity notes. [German] TT - Der elektronische mutterpass. *Gynakologe*, *39*, 278–282.	Full text could not be retrieved
