# Supplementary material for: Understanding women’s, caregivers’, and providers’ experiences with home-based records: A systematic review of qualitative studies
Source: PLoS One. 2018 Oct 4;13(10):e0204966. doi: 10.1371/journal.pone.0204966 (PMC6171900; doi:10.1371/journal.pone.0204966)
Supplement: S1 File — (PDF) [file pone.0204966.s005.pdf]

The perspectives of women, caregivers and healthcare professionals on the feasibility, acceptability, affordability, and equity considerations of Home-Based Records: A protocol for a WHO qualitative systematic review using GRADE CERQual

Olivia Magwood[1,2], Alain Mayhew[1], Victoire Kpade[1], Sandy Oliver[3], Joanna Vogel[4], Kevin Pottie[1,5]

1. *C.T. Lamont Primary Health Care Research Centre, Bruyère Research Institute, Ottawa ON*
2. *Department of Population Medicine, University of Guelph, Guelph, Canada*
3. *University College London, London, UK*
4. *Office of the Assistant Director-General, Family, Women's and Children's Health*
5. *Departments of Family Medicine & Epidemiology and Community Medicine, University of Ottawa, Canada*

BACKGROUND

Throughout history, women and children have faced discrimination in many aspects of life. Even today, women still face inequalities in terms of income, education, participation in society, and health (Rogers, 1997). Newborns and children are particularly vulnerable because they depend on adults for basic needs such as food, education, and healthcare (Rogers, 1997). Maternal, newborn and child health (MNCH) aims to improve the health and wellbeing of women, newborns, children, youth and their families. MNCH is a public health issue that will determine the health of the next generation and predict future health care systems (Barros, 2013). The development and implementation of practical health information tools and interventions are necessary to further reduce health disparities. To obtain quality healthcare, women and caregivers need to understand, accept, value, and participate in preventive interventions.

A home-based record (HBR) is a document (paper or electronic format) covering one or more components of preventive/curative antenatal, postnatal, newborn, and child health, vaccination (including Human Papillomavirus (HPV)) and nutrition that is maintained and used in the household by the woman for maternal health and/or caretakers of the household's children (Shah, 1993). The home-based record documents maternal, newborn and child health information related to health care visits and treatments received. In addition, health education messages are often included in home-based records to promote better health care seeking, healthy behaviours, and home care practices. HBRs come in different forms starting with the most basic antenatal or vaccination only cards, and progressing to vaccination plus cards, maternal and child health books, and mobile records. Mobile records enable patients to access their health information through the internet, cellular devices, and tablets. This increasing use of mobile devices to access health information reflects the increasing trend in the digitization of healthcare (Bouri, 2014).

OBJECTIVES

The objective of this project is to identify, appraise and synthesize the best available evidence of the feasibility and acceptability of home-based records from the perspectives of women, family and community members, and health providers. This summary of the evidence would then help inform policy makers in considering the use of HBRs and develop techniques that increase their value to women and their children.

METHODS

Key Question:

1. Are either single or multi domain home-based records feasible, acceptable, affordable and equitable from the perspective of women, family members, and health provider stakeholders?

Approach: The Qualitative Review

Qualitative methods allow researchers to study the complexity of healthcare systems and patient experiences (Smith, 2011). This type of study aims to explain a phenomena using collected evidence. Qualitative synthesis is a methodology that explores and interprets the meanings of these findings (Bearman, 2013). This synthesis will allow us to gain in-depth understanding of stakeholder perspectives. We will conduct a search of literature to gather primary qualitative and mixed-methods studies to draw together findings, and we will utilize a qualitative framework analysis to explore their meanings.

Study Criteria

This review will include available qualitative evidence which focuses on the implementation strategies, feasibility, acceptability, affordability and equity of HBRs. The focus of this review is on LMICs, but if no relevant evidence is available, data from high-income countries will be considered. Studies must meet the following criteria:

- Population: Women, family, and health providers.
- Intervention: Home-based record. Includes but is not limited to: vaccination only records (record of basic identifying information and immunization services received), vaccination-plus records (record of child growth and development, immunization services, and a limited set of basic information related to child survival), child health book (record of birth characteristics, health services received, growth and feeding practices, guidance to parents), pregnancy case-notes, and maternity personal health records (PHRs). These health records may be in paper form or electronic form to be considered for this review.
- Outcomes: Value of outcome, feasibility, acceptability, affordability and equity considerations.

Search Strategy

A search strategy will be developed and peer-reviewed by a librarian. The following electronic databases will be searched for qualitative and mixed-methods studies: MEDLINE, EMBASE, CINAHL, PsycINFO and ERIC. Studies will be restricted to the years 1992-2017, to reflect the gap in evidence since the latest WHO evaluation of home-based records (Shah, 1993). There will be no language restrictions set for the search. The search strategy will use a combination of indexed terms and free text words. In addition we will search grey literature for published guidelines and reports on home-based records on CDC, ECDC, and WHO websites. The literature search results will be uploaded to a reference manager software package to facilitate the study selection process.

Study Screening and Selection

Two review authors will independently assess all the potential studies identified as a result of the search strategy for inclusion. We will resolve any disagreements through discussion or, if required, we will consult a third review author. The full texts of potentially eligible citations will then be retrieved and screened independently in duplicate. Studies retrieved from Part 1 of this project may be considered for inclusion if they meet the eligibility criteria. Study selection will be verified on up to 10% of the studies by a member of the funding agency.

Data Extraction

We will develop a standardized extraction sheet informed by a framework analysis (framework: Social-Ecological Model). Teams of two reviewers will extract data in duplicate and independently. The two reviewers will compare results and resolve disagreements by discussion or with help from a third reviewer. At a minimum we will extract results as they apply to the framework analysis. Data extraction will be verified on up to 10% of the studies by a member of the funding agency.

Quality assessment of included studies

The quality of primary studies will be assessed using the Critical Appraisal Skills Programme (CASP). CASP is a tool that assesses the validity, results, and applicability of results of clinical research (Hannes, 2010).

Certainty of the evidence of included studies

We will use the Confidence in the Evidence from Reviews of Qualitative research (CERQual) tool to assess the confidence of our findings. This tool is a new method for assessing the strength of qualitative review evidence, similar to how the GRADE approach assesses the strength of quantitative evidence (Lewin, 2015). CERQual bases the evaluation on four criteria: (a) methodological limitations of included studies supporting a review finding, (b) the relevance of included studies to the review question, (c) the coherence of the review finding, and (d) the adequacy of the data contributing to a review finding.

Qualitative Analysis and Synthesis

We will use framework analysis using the Social-Ecological Model to identify and group ideas of feasibility, acceptability, affordability and equity across key populations.

DISSEMINATION OF RESULTS

We will publish this systematic review in an open access journal.

FUNDING SOURCES

This work is supported by the Japan International Cooperation Agency (JICA).

CONFLICTS OF INTEREST

There are no potential conflicts of interest to report.

REFERENCES

Barros, A. J., & Victora, C. G. (2013). Measuring Coverage in MNCH: Determining and Interpreting Inequalities in Coverage of Maternal, Newborn, and Child Health Interventions. *PLoS Medicine, 10*(5). doi:10.1371/journal.pmed.1001390

Bearman, M., & Dawson, P. (2013). Qualitative synthesis and systematic review in health professions education. *Medical Education, 47*(3), 252-260. doi:10.1111/medu.12092

Bouri, N., & Ravi, S. (2014). Going Mobile: How Mobile Personal Health Records Can Improve Health Care During Emergencies. *JMIR mhealth and uhealth, 2*(1). doi:10.2196/mhealth.3017

Hannes, K., Lockwood, C., & Pearson, A. (2010). A Comparative Analysis of Three Online Appraisal Instruments' Ability to Assess Validity in Qualitative Research. *Qualitative Health Research, 20*(12), 1736-1743. doi:10.1177/1049732310378656

Lewin, S., Glenton, C., Munthe-Kaas, H., Carlsen, B., Colvin, C. J., Gülmezoglu, M., . . . Rashidian, A. (2015). Using Qualitative Evidence in Decision Making for Health and Social Interventions: An Approach to Assess Confidence in Findings from Qualitative Evidence Syntheses (GRADE-CERQual). *PLOS Medicine, 12*(10). doi:10.1371/journal.pmed.1001895

Rogers, A. C. (1997). Vulnerability, health and health care. *Journal of Advanced Nursing, 26*(1), 65-72. doi:10.1046/j.1365-2648.1997.1997026065.x

Shah, P. M., Selwyn, B. J., Shah, K., & Kumar, V. (1993). Evaluation of the home-based maternal record: a WHO collaborative study. *Bulletin of the World Health Organization, 71*(5), 535-548.

Smith, J., & Firth, J. (2011). Qualitative data analysis: the framework approach. *Nurse Researcher, 18*(2), 52-62. doi:10.7748/nr2011.01.18.2.52.c8284
